# Effizienz von Steuerungs- und integrierten Versorgungsmodellen für wenig akute Patient:innen in der Akut- und Notfallversorgung – Ergebnisse einer prospektiven Kohortenstudie

**DOI:** 10.1007/s00103-026-04298-4

**Published:** 2026-09-18

**Authors:** Daniela Krüger, Cornelia Wäscher, Sylwia Steinke, Felix Holzinger, Hanna Winkler, Martin Möckel, Konrad Schmidt, Anna Slagman

**Affiliations:** 1 Notfall- und Akutmedizin, Campus Mitte und Virchow, Charité – Universitätsmedizin Berlin, Berlin, Deutschland; 2 Institut für Allgemeinmedizin, Charité – Universitätsmedizin Berlin, Berlin, Deutschland; 3 Institut für Sozialmedizin, Epidemiologie und Gesundheitsökonomie, Charité – Universitätsmedizin Berlin, Berlin, Deutschland; 4 Institut für Allgemeinmedizin, Medizinische Hochschule Brandenburg Theodor Fontane, Brandenburg, Deutschland

**Keywords:** Patient:innensteuerung, Notaufnahme, Wenig akute Patient:innen, Integriertes Notfallzentrum (INZ), Notfallreform, Patient streaming, Emergency department, Low-acuity patients, Integrated emergency center (IEC), Emergency care reform

## Abstract

**Einleitung:**

Gemäß der geplanten Notfallreform soll die Steuerung wenig akuter Patient:innen in ambulante Strukturen Notaufnahmen entlasten. Ziel ist der Vergleich der Versorgungseffizienz verschiedener Steuerungs- und Versorgungsmodelle am Notaufnahmestandort.

**Methoden:**

Die prospektive, multizentrische Kohortenstudie vergleicht in einer Primärdatenkohorte wenig akuter Patient:innen und einer Routinedatenkohorte unselektierter Patient:innen 3 Studienarme: 1) klinikeigene Ambulanz, 2) Notdienstpraxis der Kassenärztlichen Vereinigung (KV-Praxis), 3) Standardversorgung mit ärztlich bewertetem Steuerungspotenzial. Primärer Endpunkt: Versorgungseffizienz als Anteil der in die Alternativstruktur gesteuerten Patient:innen bzw. als Steuerungspotenzial. Sekundärer Endpunkt: Patient:innensicherheit (Notaufnahmewiedervorstellung, stationäre Aufnahme).

**Ergebnisse:**

Die Versorgungseffizienz für wenig akute Patient:innen (Primärdatenkohorte: *n* = 1096) lag für die klinikeigene Ambulanz bei 49,5 % (*n* = 145) und für die KV-Praxis bei 77,2 % (*n* = 322). Das Steuerungspotenzial lag bei 73,4 % (*n* = 240). Für unselektierte Notaufnahmepatient:innen (Routinedatenkohorte: *n* = 68.010) lag die Versorgungseffizienz der klinikeigenen Ambulanz bei 11,4 % (*n* = 2558) und für die KV-Praxis bei 29,9 % (*n* = 13.668). Eine Notaufnahmewiedervorstellung binnen 30 Tagen erfolgte nach Versorgung in der klinikeigenen Ambulanz bei 2,1 % (*n* = 1; keine stationäre Aufnahme) und in der KV-Praxis bei 10,4 % (*n* = 14; stationäre Aufnahmen: 7,5 %, *n* = 10).

**Diskussion:**

Die Steuerung und Versorgung wenig akuter Fälle am Notaufnahmestandort ist in der KV-Praxis effizienter, zeigt jedoch potenziell höhere Sicherheitsrisiken. Die Versorgungseffizienz in der Primärdatenkohorte ist rekrutierungsbedingt überschätzt, die Routinedatenkohorte bietet realistischere Werte.

**Zusatzmaterial online:**

Zusätzliche Informationen sind in der Online-Version dieses Artikels (https://doi.org/10.1007/s00103-026-04298-4) enthalten.

## Abstract (en)

**Introduction:**

According to the emergency care reform, streaming low-acuity patients to outpatient care structures seeks to relieve emergency departments (EDs). The aim was to compare the care efficiency of different streaming models at the ED site.

**Methods:**

This prospective, multicenter cohort study compared three study arms in a primary-data cohort of low-acuity patients and a routine-data cohort of unselected patients: 1) a hospital-operated outpatient practice (hospital practice), 2) an on-call practice operated by the Association of Statutory Health Insurance Physicians (SHI practice), and 3) standard care with physician-assessed streaming potential. The primary endpoint was care efficiency: the proportion of patients directed to the alternative care structure or the assessed streaming potential. The secondary endpoint was patient safety (ED revisit, hospital admission).

**Results:**

Among low-acuity patients (primary-data cohort: *n* = 1096), care efficiency was 49.5% (*n* = 145) for the hospital practice and 77.2% (*n* = 322) for the SHI practice. The physician-assessed streaming potential was 73.4% (*n* = 240). Among unselected ED patients (routine-data cohort: *n* = 68.010), care efficiency was 11.4% (*n* = 2558) for the hospital practice and 29.9% (*n* = 13.668) for the SHI practice. An ED revisit within 30 days occurred in 2.1% (*n* = 1) after treatment in the hospital practice (no hospital admissions) and in 10.4% (*n* = 14) after treatment in the SHI practice (hospital admissions: 7.5%, *n* = 10).

**Discussion:**

The streaming and care of low-acuity patients at the ED site was more efficient in the SHI practice but was associated with potentially greater safety risks. Care efficiency in the primary-data cohort is likely overestimated due to recruitment, whereas the routine-data cohort provides more realistic estimates.

## Einleitung

Die Steuerung wenig akuter Patient:innen in integrierte ambulante Versorgungsstrukturen soll Notaufnahmen entlasten. Seit vielen Jahren diskutiert und in Pilotprojekten erprobt [1], hält dieser Ansatz Einzug in den Gesetzesentwurf der Bundesregierung zur Reform der Notfallversorgung [2]: Als Modell soll das sogenannte Integrierte Notfallzentrum (INZ) dienen [2§ 123]. Dieses ist als sektorenübergreifende Versorgungsstruktur konzipiert und soll eine enge Kooperation zwischen Notaufnahme und Notdienstpraxis der Kassenärztlichen Vereinigung (KV) am Standort sowie optional eine Zusammenarbeit mit Partnerpraxen umfassen [2§ 123]. Richtlinien zur sachlichen und personellen Ausgestaltung sollen vom Gemeinsamen Bundesausschuss (G-BA) formuliert werden [2§ 123c].

Obwohl erste Empfehlungen zur INZ-Ausgestaltung entwickelt wurden [3], sind Versorgungseffizienz und Gesamtwirkung der konkreten Umsetzung der unterschiedlichen Versorgungsmodelle noch unklar. Monozentrische Pilotstudien verschiedener Versorgungsmodelle aus Deutschland weisen zwar auf ein relevantes Entlastungspotenzial für Notaufnahmen bei wenig akuten Patient:innen hin [1, 4–7]; systematische Vergleiche zur Versorgungseffizienz unterschiedlicher Modelle innerhalb eines Studiendesigns unter Berücksichtigung der konkreten Modellumsetzung liegen bislang jedoch nicht vor.

Ziel dieses Beitrags ist ein systematischer, mehrdimensionaler Vergleich der Versorgungseffizienz und der Gesamtwirkung zweier integrierter Steuerungs- und Versorgungsmodelle am Notaufnahmestandort mit der Standardversorgung in der Notaufnahme unter Einbezug qualitativer Perspektiven auf Struktur- und Prozessmerkmale. Die Gesamtwirkung wurde dabei anhand des „Makro-Meso-Mikro-Modells“ der organisationsbezogenen Versorgungsforschung als mehrdimensionales Konzept operationalisiert [8, 9]. Die Datenbasis dazu lieferte die prospektive, multizentrische Kohortenstudie „Patient:innen-Navigation in der deutschen Notfallversorgung“ (NODE) an 6 Notaufnahmen in Berlin.

Primäres Untersuchungsziel war die primäre Versorgungseffizienz der Modelle, verstanden als tatsächliche bzw. potenzielle Entlastung der Notaufnahme. Diese entsprach dem Anteil der Patient:innen, die initial in eine alternative Versorgungsstruktur gesteuert wurden bzw. nach ärztlicher Einschätzung hätten gesteuert werden können. Verglichen wurden ein klinikeigenes integriertes Versorgungsmodell (Notaufnahme und klinikeigene Ambulanz) und ein Kooperationsmodell (Notaufnahme und KV-Notdienstpraxis). Ein in der Standardversorgung ermitteltes Steuerungspotenzial wurde gegenübergestellt. Sekundäre Untersuchungsziele betreffen u. a. Prozessqualität (Ressourcenverbrauch, Prozesszeiten), Kosten, Patient:innensicherheit (Mortalität, Wiedervorstellung Notaufnahme, stationäre Aufnahme) und Patient:innenzufriedenheit [10]. Die Untersuchungspopulation umfasste wenig akute bzw. sich selbstständig in der Notaufnahme vorstellende Patient:innen ohne unmittelbaren notfallmedizinischen Behandlungsbedarf.

Zu den im Folgenden dargestellten Methoden und Ergebnissen steht umfangreiches Onlinematerial zur Verfügung, darunter „E-Tabellen“ und „E-Abbildungen“, auf die im Text verwiesen wird.

## Methoden

### Studiendesign und Setting.

Dieser Beitrag berichtet Analysen einer konvergenten Mixed-Methods-Studie [10]. Das zentrale Modul war eine prospektive, multizentrische Kohortenstudie mit 3 Studienarmen an 6 Notaufnahmestandorten in Berlin:

- Arm 1: klinikeigenes ambulantes Versorgungsmodell am Standort der Notaufnahme („klinikeigene Ambulanz“),
- Arm 2: Notdienstpraxis der Kassenärztlichen Vereinigung am Standort der Notaufnahme („KV-Notdienstpraxis“),
- Arm 3: Notaufnahme mit Standardversorgung und ärztliche Einschätzung zur Versorgungsebene der Patient:innen („Standardversorgung mit ärztlicher Einschätzung“).

Die Rekrutierung für die Primärdatenkohorte erfolgte vom 01.06.2023 bis zum 31.08.2024 zu den Öffnungszeiten der Alternativstruktur (E-Abbildung 1). Eingeschlossen wurden volljährige, einwilligungsfähige, fußläufige Selbstvorsteller:innen, die keiner unmittelbaren medizinischen Versorgung bedurften (fortlaufend als „wenig akut“ bezeichnet, [11]) sowie über ausreichende Deutsch- oder Englischkenntnisse verfügten. Ausgeschlossen wurden Patient:innen, die sich gezielt in der alternativen ambulanten Versorgungsstruktur vorstellten.

Ergänzt wurde die prospektive Kohortenstudie durch Routinedatenanalyse aller unselektierten, volljährigen Notaufnahmepatient:innen an allen beteiligten Studienzentren sowie einer Selektion von fußläufigen Selbstvorstellungen zur Vergleichbarkeit mit der Studienpopulation (Annahme: 45 % aller Notaufnahmepatient:innen, [12]). Zudem wurden qualitative Prozessbeobachtungen durchgeführt, die zur Ergebnistriangulation herangezogen wurden.

### Studienpopulation und Studienablauf.

Die Patient:innen wurden nach Triagierung und Steuerung in das jeweilige Versorgungssetting (tatsächliche Steuerung, Arm 1 und 2) und in der Regel vor Behandlungsbeginn rekrutiert. Die ärztliche Einschätzung der Versorgungsebene (Studienarm 3) wurde mittels Bewertungsbogen [13] dokumentiert und sollte nach ärztlicher Anamnese sowie vor Vorliegen diagnostischer Befunde erfolgen (E-Abbildung 2). Hierbei standen für die ärztliche Bewertung als Versorgungsebenen zur Auswahl:

- Versorgung in einer Praxis möglich („ambulante Praxis“),
- Versorgung in der Notaufnahme erforderlich („Notaufnahme“).

Nach Behandlungsende wurden die Routinedaten zum klinischen Verlauf aller eingeschlossenen Patient:innen extrahiert und in einen elektronischen Prüfbogen (Electronic Case Report Form, eCRF) übertragen. 30 Tage nach Vorstellung in der Notaufnahme wurde ein Follow-up durchgeführt. Nach Abschluss der Rekrutierung erfolgte die Extraktion von Routinedaten zum Versorgungsverlauf aller volljährigen Patient:innen der teilnehmenden Notaufnahmen für den Rekrutierungszeitraum.

Qualitative Prozessbeobachtungen mithilfe eines standardisierten Protokolls (E-Tabelle 1) begleiteten die Rekrutierung der Kohortenstudie an 2–3 Tagen zur explorativen Prozess- und Strukturcharakterisierung.

### Primäres und sekundäre Untersuchungsziele.

Primäres Untersuchungsziel war die primäre Versorgungseffizienz der Modelle, gemessen am Anteil von Patient:innen, welche statt in der Notaufnahme in einer alternativen Versorgungsstruktur behandelt wurden (Studienarm 1 und 2) bzw. nach ärztlicher Einschätzung potenziell behandelt werden könnten (Studienarm 3). Die Analyse erfolgt auf Basis zweier Grundgesamtheiten und Datengrundlagen (E‑Tabelle 2).

Aufgrund unvollständiger Routinedaten wurden teilweise Proxys zur Analyse der primären Versorgungseffizienz herangezogen (E-Tabelle 3).

Sekundäre Untersuchungsziele umfassten die Patient:innensicherheit (Mortalität und stationäre Aufnahme am Vorstellungstag sowie Notaufnahmewiedervorstellung und stationäre Aufnahme im 30-Tages-Nachbeobachtungszeitraum, inklusive Aufnahme auf die Intensivstation), die sekundäre Versorgungseffizienz (Anteil der ausschließlich in einer Alternativstruktur versorgten Patient:innen), den Ressourcenverbrauch, die Kosten für Konsile und Diagnostik in der Notaufnahme aus Perspektive des Krankenhauses (am Vorstellungstag) und die Kosten aus gesellschaftlicher Perspektive (im 30-Tages-Nachbeobachtungszeitraum) sowie die Patient:innenzufriedenheit (E-Tabelle 4).

### Statistische Analyse.

Die Auswertung des primären Endpunkts erfolgte mittels Chi^2^-Tests und binomialer 95 %-Konfidenzintervalle auf Basis der Primärdaten- und Routinedatenkohorte. Die Fallzahlplanung erfolgte auf Basis der wissenschaftlichen Literatur ([14–26]; Annahmen: E‑Tabelle 5). Diese wurde in Bezug zur selektierten und unselektierten Grundgesamtheit gesetzt.

Die Beschreibung der Studienpopulation sowie die Analyse der sekundären Endpunkte erfolgten explorativ mittels deskriptiver Statistik (SPSS-Version 30; IBM, Armonk, NY, USA). Für die Bestimmung der Kosten am Vorstellungstag wurde der Ressourcenverbrauch in der Notaufnahme anhand von Standardkostensätzen bewertet. Die Kosten der Notaufnahmediagnostik wurden auf Grundlage des Nebenkostentarifs der Deutschen Krankenhausgesellschaft (DKG-NT) berechnet [27]. Für die gesellschaftlichen Kosten im 30-Tage-Nachbeobachtungszeitraum wurden Kostensätze nach Muntendorf et al. [28] verwendet.

**Abb. 1:**
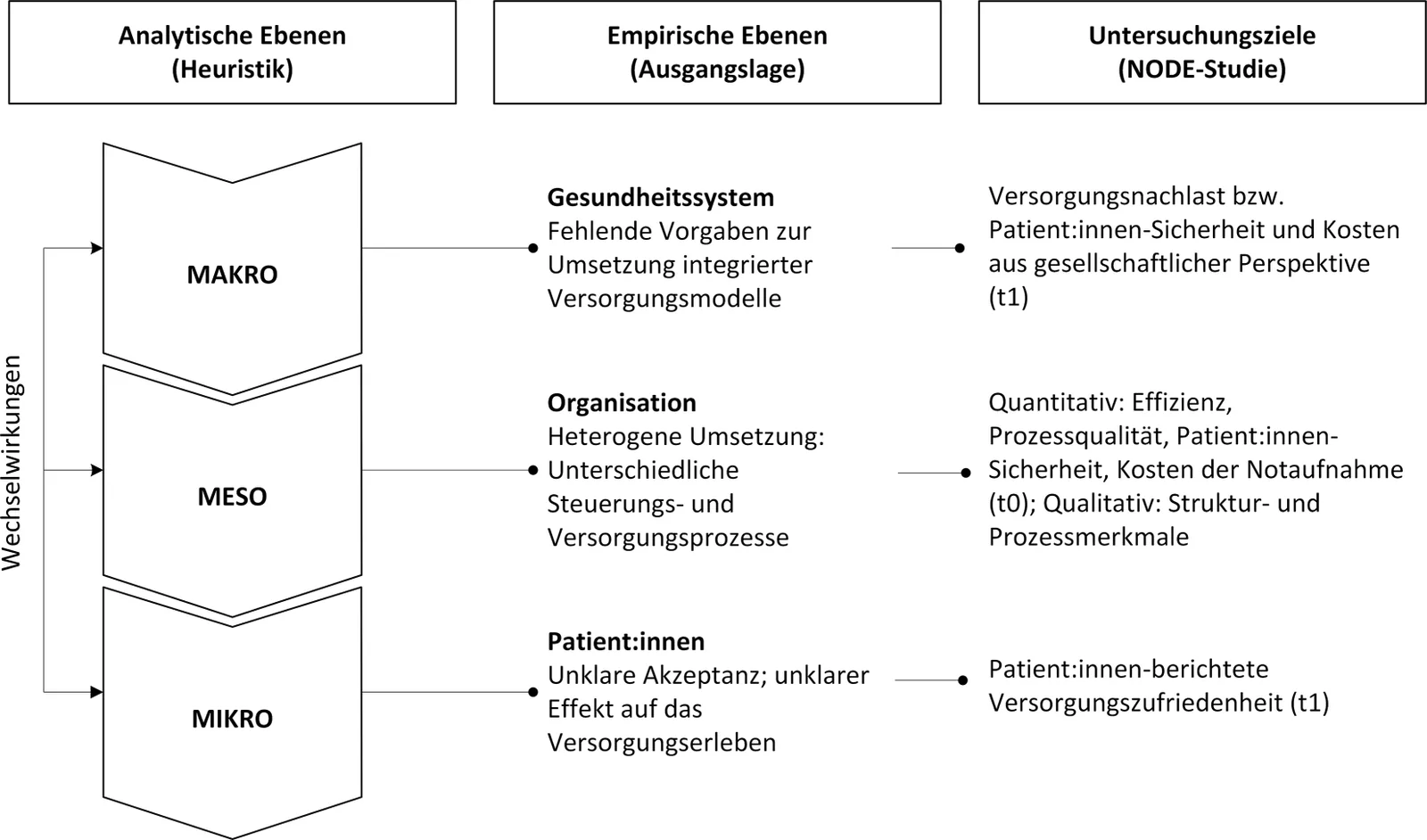
Makro-Meso-Mikro-Modell mit Wechselwirkungen zur Beurteilung der Gesamtwirkung von Versorgungsmodellen in der Studie „Patient:innen-Navigation in der deutschen Notfallversorgung“ (NODE)

Die Beobachtungsprotokolle wurden einer qualitativen Inhaltsanalyse mittels MAXQDA (MAXQDA-Version 24; VERBI – Software. Consult. Sozialforschung GmbH, Berlin, Deutschland) unterzogen [29]. Das „Makro-Meso-Mikro-Modell“ diente als Heuristik zur Strukturierung der Studienziele und der Ergebnisse (Abb. 1).

### Datenerfassung.

Die Datenerfassung der Primärdatenkohorte erfolgte mithilfe von *Research Electronic Data Capture* (REDCap; Vanderbilt University, Nashville, TN, USA), in der Routinedatenkohorte als CSV-Excel-Dateien (Microsoft Corporation, Redmond, WA, USA). Semistrukturierte Beobachtungsprotokolle wurden digitalisiert.

### Regulatorik.

Die Studie wurde von der Ethikkommission der Charité – Universitätsmedizin Berlin (EA2/153/22; Datenschutz-Nr. 22-0771) geprüft und bewilligt. Die Studie ist im Deutschen Register für Klinische Studien registriert (DRKS00030398).

## Ergebnisse

**Abb. 2:**
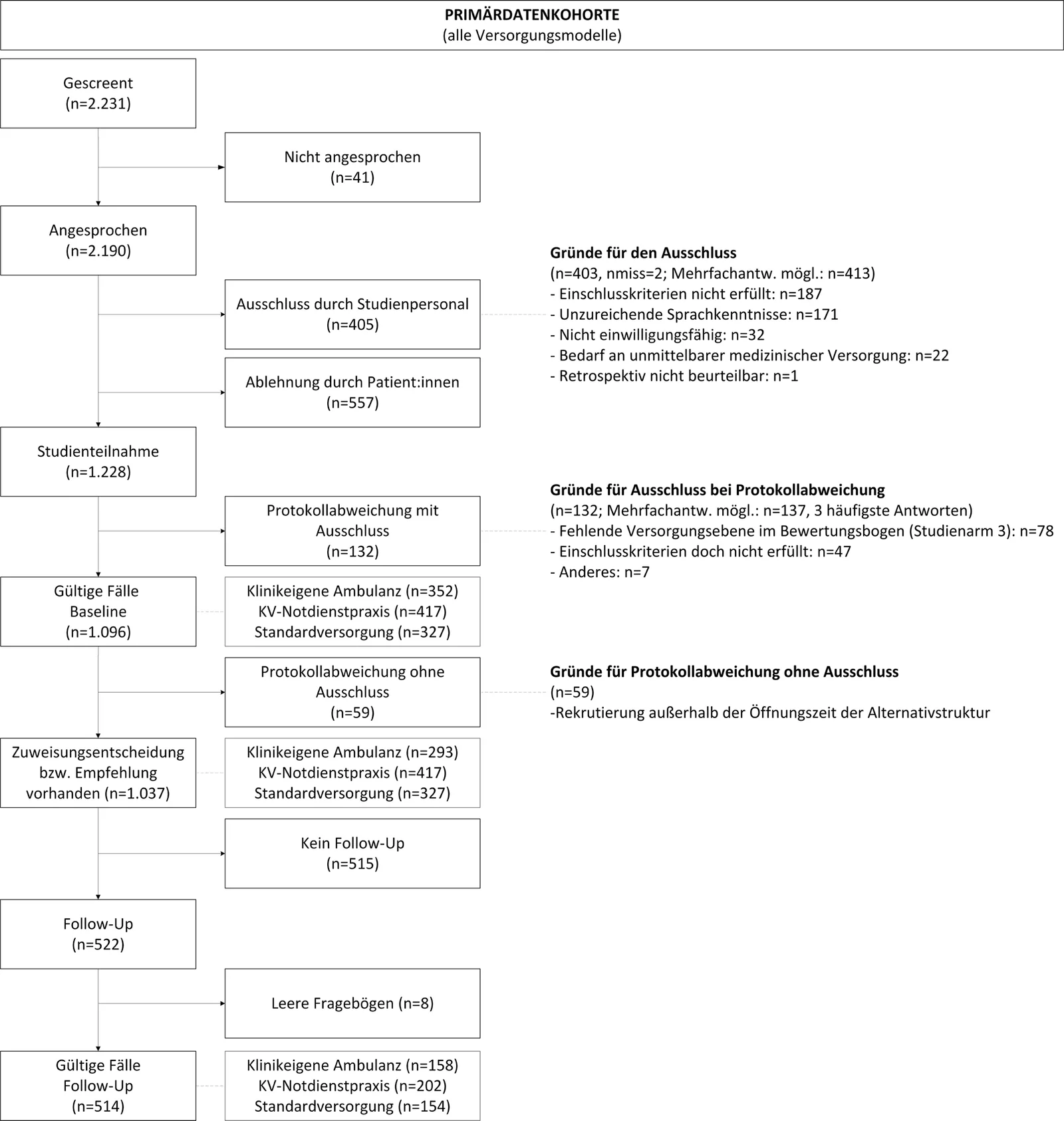
Patient:innen-Fluss der Primärdatenkohorte der prospektiven, multizentrischen Mixed-Methods-Kohortenstudie NODE. (Dargestellt werden Ein- und Ausschlüsse, Protokollabweichungen sowie die Fallzahlen der 3 Studienarme (Studienarm 1: klinikeigene Ambulanz, Studienarm 2: KV-Notdienstpraxis, Studienarm 3: Standardversorgung mit ärztlicher Einschätzung). Fallzahlen basieren auf einer Baselinebefragung am Vorstellungstag sowie einem Follow-up nach 30 Tagen. *KV* Kassenärztliche Vereinigung, *n* Fallzahl)

Für die prospektive Kohortenstudie wurden 2231 Patient:innen gescreent und 1228 eingeschlossen. Die finale Studienpopulation zur Baseline bestand aus 1096 Patient:innen (Abb. 2). Das mediane Alter der Studienpopulation betrug 39 Jahre (SD = 14 Jahre), 53 % (*n* = 573) der Teilnehmenden waren weiblich, 46 % (*n* = 499) männlich und 1 % (*n* = 8) divers. Eine Basischarakterisierung der Studienpopulation und der ausgeschlossenen Patient:innen ist in den E‑Tabellen 6 und 7 zu finden.

Der primäre Endpunkt lag für 95 % (*n* = 1037) der Fälle vor (Abb. 2). 47 % der Patient:innen (*n* = 514) wurden im 30-Tage-Follow-up befragt (E-Tabelle 8).

Für die beiden Steuerungsmodelle (Arm 1, 2) lagen Routinedaten aus 4 Studienzentren für *n* = 244.843 Fälle vor. Davon flossen 28 % (*n* = 68.010) in die Bewertung des primären Endpunktes ein (E-Abbildung 3). Eine Basischarakterisierung der Routinedatenkohorte befindet sich in E‑Tabelle 9.

### Mesoebene: Prozessmerkmale, Versorgungseffizienz, Ressourcennutzung und Kosten der Notaufnahmeversorgung

Auf der Struktur- und Prozessebene^1^ finden sich Unterschiede zwischen und innerhalb der beiden Studienarme mit Steuerungsmodell (E-Tabelle 10). Eine zentrale Steuerung ist in 2 von 4 Standorten mit Steuerungsmodell implementiert. An 2 Standorten erfolgt die Steuerung auf Grundlage einer standardisierten Verfahrensanweisung (SOP) bzw. eines internen Schemas unter Berücksichtigung des Behandlungsspektrums der Versorger:innen sowie der Triagedokumentation. An 3 der 4 Standorte erfolgt die Steuerung hauptsächlich durch Pflegekräfte, an einem Standort durch Pflegekräfte und Ärzt:innen.

Die Zielpopulation der Alternativversorgungsstruktur ist an 2 Standorten enger definiert. Eine sektorale Trennung der Ressourcen zwischen Notaufnahme und Alternativstruktur besteht an 3 der 4 Standorte (E-Tabelle 10).

Im Hinblick auf die Versorgungseffizienz zeigten sich deutliche Unterschiede zwischen den Versorgungsmodellen (Tab. 1).^2^ In Arm 1 der Routinedatenkohorte wurden 25,3 % (*n* = 2558) der sich selbstständig in der Notaufnahme vorstellenden Patient:innen initial der klinikeigenen Ambulanz zugewiesen. Davon wurden 64,8 % (*n* = 725)^3^ fallabschließend versorgt. In Arm 2 wurden demgegenüber 66,7 % (*n* = 13.668) der KV-Notdienstpraxis zugewiesen und davon 91,8 % (*n* = 5618)^4^ fallabschließend versorgt.

**Tab. 1:** Primäre und sekundäre Versorgungseffizienz bzw. Steuerungspotenzial in der prospektiven, multizentrischen Mixed-Methods-Kohortenstudie NODE

Grundgesamtheit	Endpunkt		Studienarm 1:	Studienarm 2:	*P‑Wert*^b^	Studienarm 3:	*P‑Wert*^c^
			Klinikeigene Ambulanz	KV-Notdienstpraxis^a^		Standardversorgung mit ärztlicher Einschätzung	
Wenig akute Patient:innen der Routinedatenkohorte^d^	Stichprobengröße	*n*	10.112	20.493	–	–	–
	Theoretische Annahme^e^	*%*	30 %	50 %	–	–	–
	Primäre Effizienz – initiale Zuweisung^f^	*% (n)*	25,3 (2558)	66,7 (13.668)	*<0,001*	–	–
		*95%-KI*	24,5; 26,2	66,0; 67,3	–	–	–
Alle Notaufnahmepatient:innen der Routinedatenkohorte	Stichprobengröße	*n*	22.470	45.540	–	–	–
	Theoretische Annahme^e^	*%*	14 %	23 %	–	–	–
	Primäre Effizienz – initiale Zuweisung^f^	*% (n)*	11,4 (2558)	29,9 (13.668)	*<0,001*	–	–
		*95%-KI*	11,0; 11,8	29,6; 30,4	–	–	–
	Sekundäre Effizienz – fallabschließende Behandlung^g,h^	*% (n)*	64,8 (725)	91,8 (5618)	–	–	–
		*95%-KI*	62,2; 67,6	91,1; 92,5	–	–	–
Wenig akute Patient:innen der Primärdatenkohorte	Stichprobengröße	*n*	293^i^	417	–	327	–
	Theoretische Annahme^e^	*%*	30 %	50 %	–	70 %	–
	Primäre Effizienz – initiale Zuweisung	*% (n)*	49,5 (145)	77,2 (322)	n.v.	73,4 (240)	*<0,001*
		*95%-KI*	43,8; 55,2	73,0; 81,0	–	68,4; 78,0	–
	Sekundäre Effizienz – fallabschließende Behandlung^g^	*% (n)*	80,7 (117)	94,4 (304)	–	–	–
		*95%-KI*	73,7; 86,5	91,5; 96,5	–	–	–

Darstellung in Bezug auf die initiale Zuweisung in die Alternativstruktur (tatsächliche Steuerung: Studienarm 1 und 2) bzw. Empfehlung für die Alternativstruktur (Steuerungspotenzial: Studienarm 3).

^a^Für ein Studienzentrum mit KV-Notdienstpraxis lagen in der Routinedatenkohorte Behandlungszahlen quartalsweise vor und wurden an den Rekrutierungszeitraum der Studie angepasst (siehe E‑Tabelle 3).

^b^Pearson-Chi^2^-Test zwischen Studienarm 1 und 2.

^c^Pearson-Chi^2^-Test zwischen den Studienarmen 1, 2 und 3.

^d^Proxy: Anteil der Selbstvorstellungen = 45 % an allen Notaufnahmepatient:innen [12].

^e^Annahmen siehe E‑Tabelle 5.

^f^Pooled Proportions.

^g^Abschließend in der Versorgungsstruktur behandelte Patient:innen als Anteil an allen in der jeweiligen alternativen Versorgungsstruktur behandelten Patient:innen.

^h^Behandlungszahlen nur für je ein Zentrum verfügbar.

^i^Fallzahl abzüglich *n* = 59 Fälle bei Nichtverfügbarkeit der Alternativstruktur rekrutiert.

*95%-KI* 95 %-Konfidenzintervall, *n.v.* nicht vorgesehen, *n* Fallzahl, *%* relativer Anteil

Die Zuweisungsanteile in der Primärdatenkohorte der selbstständigen Vorstellungen lagen aufgrund einer Überrekrutierung in den alternativen Versorgungsstrukturen höher: 49,5 % (*n* = 145) für die klinikeigene Ambulanz (davon fallabschließend: 80,7 %, *n* = 117) sowie 77,2 % (*n* = 322) für die KV-Notdienstpraxis (davon fallabschließend: 94,4 %, *n* = 304). In Arm 3 erhielten 73,4 % (*n* = 240) die ärztliche Einschätzung, sie seien ambulant behandelbar.

Basierend auf der Routinedaten- und Primärdatenkohorte zeigte das Modell der KV-Notdienstpraxis somit eine signifikant höhere primäre und eine höhere sekundäre Versorgungseffizienz (Tab. 1).

Unterschiede im Ausmaß der sekundären Versorgungseffizienz fanden sich im Ressourcenverbrauch für die Notaufnahme wieder: Nach initialer Zuweisung in die klinikeigene Ambulanz (Studienarm 1) bzw. Einschätzung zur ambulanten Versorgung (Studienarm 3) wurden in der Primärdatenkohorte im hohen Maße diagnostische Leistungen oder ärztliche Konsile in Anspruch genommen (Tab. 2). Strukturell wird der Zugriff im Modell der klinikeigenen Ambulanz an einem Standort durch deren Integration in die Notaufnahme ermöglicht (E-Tabelle 10). Im Modell der KV-Notdienstpraxis ist der Zugriff eingeschränkt (E-Tabelle 10); es erfolgt eine ressourcenschonendere Versorgung (Tab. 2).

**Tab. 2:** Ressourcengebrauch in der Notaufnahme und stationäre Aufnahme zur Baseline in der prospektiven, multizentrischen Mixed-Methods-Kohortenstudie NODE

% (*n*)	Studienarm 1:		Studienarm 2:		Studienarm 3:	
	Klinikeigene Ambulanz		KV-Notdienstpraxis		Standardversorgung	
	Initiale Zuweisung		Initiale Zuweisung		Ärztliche Einschätzung	
	Klinikeigene Ambulanz (*n* = 145)	NA (*n* = 148)	KV-Notdienstpraxis (*n* = 322)	NA (*n* = 95)	Ambulante Praxis (*n* = 240)	NA (*n* = 87)
Labor	53,1 (77)	52,0 (77)	4,0 (13)	32,6 (31)	43,8 (105)	74,7 (65)
Röntgen	4,8 (7)	27,0 (40)	1,6 (5)	17,9 (17)	17,1 (41)	26,4 (23)
CT	6,9 (10)	7,4 (11)	< 5	10,5 (10)	2,1 (5)	19,5 (17)
Ultraschall	16,6 (24)	25,7 (38)	2,2 (7)	9,5 (9)	17,5 (42)	31,0 (27)
EKG	11,7 (17)	13,5 (20)	< 5	20,0 (19)	15,8 (38)	28,7 (25)
Konsil	13,8 (20)	14,2 (21)	< 5	6,3 (6)	5,4 (13)	18,4 (16)
Stationär	6,2 (9)	9,5 (14)	< 5	7,4 (7)	2,1 (5)	28,7 (25)

Darstellung in Bezug auf die initiale Zuweisung in die Alternativstruktur oder Notaufnahme bei tatsächlicher Steuerung (Studienarm 1 und 2) bzw. nach ärztlicher Einschätzung mit Empfehlung zur Versorgung in einer ambulanten Praxis oder der Notaufnahme (Steuerungspotenzial: Studienarm 3); Fallzahl abzüglich *n* = 59 Fälle, die bei Nichtverfügbarkeit der Alternativstruktur rekrutiert wurden. Stationäre Aufnahmen: 6 % (*n* = 62; Fallzahl insgesamt *n* = 1035; *n*miss = 61). Aus Gründen des Datenschutzes wurde die in einem Setting durchgeführte Magnetresonanztomographie nicht berichtet.

*Labor* Blutentnahme, Blutgasanalyse, Urinuntersuchung, *NA* Notaufnahme, *KV* Kassenärztliche Vereinigung, *CT* Computertomographie, *EKG* Elektrokardiogramm, *<5* Fallzahl unter 5 Angaben aus Gründen des Datenschutzes nicht berichtet, *n* Fallzahl

Analog zum Ressourcenverbrauch unterscheiden sich auch die aus Notaufnahmeperspektive berechneten durchschnittlichen Kosten pro Patient:in für die Diagnostik und die Konsile in der Notaufnahme zwischen den Studienarmen. Nach initialer Zuweisung in die klinikeigene Ambulanz ergeben sich mit 35 € pro Patient:in die höchsten Kosten. Für die ärztliche Einschätzung zur ambulanten Versorgung (Arm 3) lagen die durchschnittlichen Kosten bei 21 € pro Patient:in. Demgegenüber fielen die Behandlungskosten im Modell der KV-Notdienstpraxis mit durchschnittlich 3 € pro Patient:in deutlich geringer aus (E-Tabelle 11).

Der Anteil stationärer Aufnahmen zur Baseline war in der KV-Notdienstpraxis (*n* < 5) geringer als in der klinikeigenen Ambulanz (6,2 %, *n* = 9; Tab. 2). Von allen Aufnahmen entfielen 3,2 % (*n* = 2) auf die Intensivstation. Es traten in keinem Studienarm Todesfälle auf.

Ein weiterer Unterschied zwischen den Versorgungsmodellen zeigte sich in der Wartezeit bis zum ersten ärztlichen Kontakt: Die mediane Wartezeit betrug in der klinikeigenen Ambulanz 100 min (IQR 47,5–162,8) gegenüber 43 min (IQR 27,0–76,5) in der KV-Notdienstpraxis (E-Abbildung 4). Patient:innen mit ärztlicher Einschätzung für die ambulante Versorgung warteten im Arm 3 im Median 79 min (IQR 39,0–126,0).

Prozessbeobachtungen und Routinedatenanalysen dokumentierten eine initiale Öffnung mit wiederholten, verlaufsbedingten Schließungen der Alternativstruktur eines Studienzentrums mit klinikeigener Ambulanz infolge einer Personalunion mit dem Notaufnahmepersonal (E-Tabelle 10; E‑Tabelle 12).

### Mikroebene: Zufriedenheit der Patient:innen mit der Versorgung

Die Patient:innenzufriedenheit (ZUF‑8, Skala = 8–32, [30]) mit der Versorgung in der jeweiligen Alternativstruktur lag im Follow-up insgesamt auf hohem Niveau (Mittelwert = 25; *n* = 451) und für beide alternativen Modelle nahezu gleichauf (klinikeigene Ambulanz, Mittelwert = 25, 95 %-Konfidenzintervall = 23–27; KV-Notdienstpraxis, Mittelwert = 25, 95 %-Konfidenzintervall = 24–26; E‑Abbildung 5).

### Makroebene: Notaufnahmewiedervorstellungen, stationäre Aufnahmen und Kosten aus gesellschaftlicher Perspektive

**Abb. 3:**
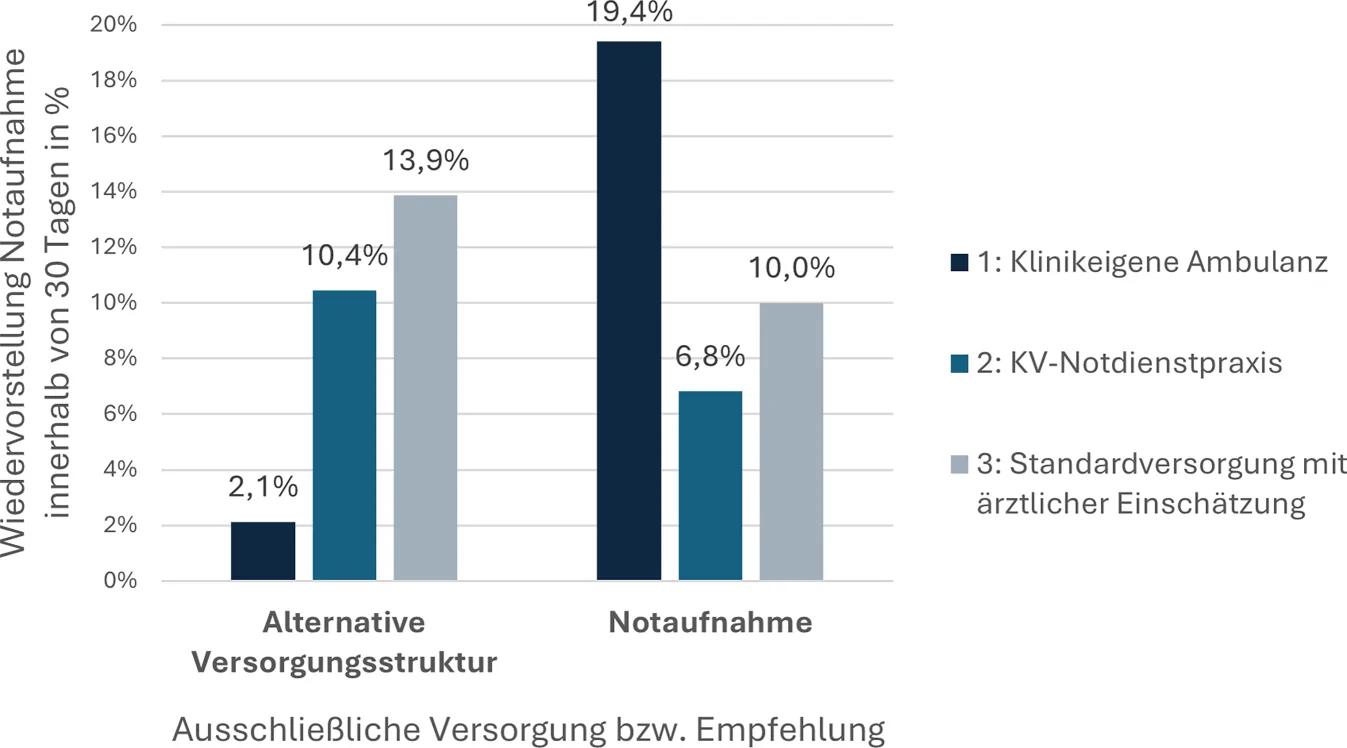
Wiedervorstellungen in der Notaufnahme innerhalb von 30 Tagen nach der Baselineversorgung in der prospektiven, multizentrischen Mixed-Methods-Kohortenstudie NODE. (Anteil der Wiedervorstellungen insgesamt: 11,3 % (*n* = 49; *N* = 433; *n*miss = 81); 81,6 % der Wiedervorstellungen in Bezug auf aktuelle Beschwerden (*n* = 40); 83,7 % ungeplant (*n* = 41). Darstellung in Bezug auf die ausschließliche Baselineversorgung in der Alternativstruktur oder der Notaufnahme sowie bei ärztlicher Empfehlung zur Versorgung in der Alternativstruktur oder der Notaufnahme. *KV* Kassenärztliche Vereinigung, *Baselineversorgung* Versorgung am Tag der initialen Vorstellung in der Notaufnahme und Studieneinschluss (t0))

Für die KV-Notdienstpraxis zeigte sich mit rund 10,4 % (*n* = 14) Wiedervorstellungen in der Notaufnahme (in der Hauptsache auf die aktuellen Beschwerden bezogen und ungeplant) eine höhere Versorgungsnachlast als für das Modell der klinikeigenen Ambulanz mit rund 2,1 % (*n* = 1; Abb. 3).^5^

Der Anteil der Patient:innen, die insgesamt im 30-Tage-Nachbeobachtungszeitraum stationär aufgenommen wurden, lag mit 7,5 % (*n* = 10) höher bei Patient:innen, die im Modell der KV-Notdienstpraxis behandelt wurden als in der klinikeigenen Ambulanz (kein Fall beobachtet; E‑Abbildung 6). Zwei der Aufnahmen (7,7 %) entfielen auf die Intensivstation. Im Zuge der Non-Response-Ermittlung für die Nachbefragung wurden zudem zwei Todesfälle erfasst (0,4 %).

Die durchschnittlichen Gesamtkosten aus gesellschaftlicher Perspektive^6^ umfassen sowohl direkte Versorgungskosten als auch indirekte Kosten, die durch den Ausfall an Bruttowertschöpfung infolge von Arbeitsunfähigkeit entstehen. Diese betrugen im 30-Tage-Nachbeobachtungszeitraum für Patient:innen, die im Modell der KV-Notdienstpraxis behandelt wurden, mit 1889 € den höchsten Wert. Im Vergleich dazu lagen die durchschnittlichen Kosten für Patient:innen, die in der klinikeigenen Ambulanz behandelt wurden, bei 1153 €. In allen Studienarmen stellten die indirekten Kosten – gemessen als Ausfall an Bruttowertschöpfung infolge von Arbeitsunfähigkeit – den größten Kostentreiber dar. Bei den durchschnittlichen Versorgungskosten (direkte Kosten) zeigte sich, dass die Patient:innen im Modell mit KV-Notdienstpraxis mit 638 € die höchsten Kosten aufwiesen. Diese wurden hauptsächlich durch die Kosten für stationäre Behandlungen verursacht (535 €). Im Modell der klinikeigenen Ambulanz und in den Studienzentren mit Standardversorgung und ärztlicher Empfehlung zur ambulanten Versorgung (Arm 3) wurden hingegen deutlich geringere durchschnittliche Versorgungskosten von 304 € bzw. 215 € pro Patient:in ermittelt (E-Tabelle 13).

## Diskussion

### Bewertung und Kontextualisierung der Versorgungseffizienz

#### Zwischen Entlastung der Notaufnahmen und Verlagerung von Versorgungsaufwand und Kosten auf Gesundheitssystem und Gesellschaft

Die primäre Versorgungseffizienz auf Basis von Routinedaten für unselektierte Notaufnahmepatient:innen lag in der vorliegenden Studie bei 11,4 % für die klinikeigene Ambulanz und bei 29,9 % für die KV-Notdienstpraxis. Die Ergebnisse liegen damit im Bereich der Annahmen der Fallzahlplanung (E-Tabelle 5). Im Vergleich zu einer in NODE durchgeführten deutschlandweiten Befragung von Notaufnahmeleitungen, die aggregierte Entlastungspotenziale ermittelte, liegt der Wert der klinikeigenen Ambulanz im unteren Bereich klinikeigener Strukturen (Median: 21 %; 25. Perzentil: 13 %; 75. Perzentil: 24 %), während die KV-Notdienstpraxis vergleichsweise hohe Werte aufweist (Median 22 %; 25. Perzentil: 13 %; 75. Perzentil: 31 %; [1]).

Die geringe Versorgungseffizienz der klinikeigenen Ambulanz ist dabei vor dem Hintergrund der eingeschränkten Verfügbarkeit und unstandardisierten Zuweisung an einem Studienzentrum zumindest teilweise als Zentrumseffekt zu interpretieren. Trotzdem stützen Studien zu einzelnen Pilotprojekten sowie das in NODE durchgeführte systematische Review die Tendenz der vergleichsweise höheren Versorgungseffizienz von Versorgungsstrukturen mit hausärztlich-allgemeinmedizinischem Patient:innenmanagement vs. alternativen notfallmedizinischen Versorgungspfaden, wenngleich mit unterschiedlichen Bezugsgrößen und entsprechend variierenden Versorgungsanteilen [4, 5, 31, 32].

Die primäre Versorgungseffizienz der Primärdatenkohorte der wenig akuten Patient:innen lag deutlich über den Werten der Routinedaten und der bestehenden Literatur und ist wegen einer Überrekrutierung in der alternativen Versorgungsstruktur methodisch bedingt überschätzt.

Hinsichtlich der sekundären Versorgungseffizienz zeigt die KV-Notdienstpraxis sowohl in der Routine- als auch in der Primärdatenkohorte deutlich höhere Anteile fallabschließend versorgter Patient:innen im Vergleich zur klinikeigenen Ambulanz. Korrespondierend hierzu sind der Ressourcenverbrauch und die Kosten für die Notaufnahmeversorgung für initial in die KV-Notdienstpraxis gesteuerte Patient:innen geringer.

Die in NODE durchgeführte deutschlandweite Befragung zeigte, dass 57 % aller Notaufnahmeleitungen mit integrierten Versorgungsmodellen Patient:innen unstandardisiert steuern [1]. Auch in dieser Studie erfolgte die Steuerung nur an 2 von 4 Standorten standardisiert. Die im Vergleich zu den Primärdaten geringere sekundäre Versorgungseffizienz in den Routinedaten der klinikeigenen Ambulanz könnte teilweise auf eine unstandardisierte Zuweisung und eine unzureichende Abstimmung des Ressourcenbedarfs in dieser Alternativstruktur zurückzuführen sein. Dies könnte zu einem erhöhten Bedarf an diagnostischer Abklärung und insgesamt zur Verlangsamung der Prozesse sowohl innerhalb der klinikeigenen Ambulanz als auch in der Notaufnahme führen, was sich indirekt in längeren Wartezeiten bis zum ersten ärztlichen Kontakt widerspiegelt.

Darüber hinaus könnten Unterschiede im Case-Mix bzw. in der Morbiditätsstruktur der Patient:innenkollektive zum unterschiedlichen Ressourcenverbrauch beigetragen haben, da das Modell im Gegensatz zur KV-Notdienstpraxis auch während der regulären Sprechzeiten geöffnet war (E-Tabelle 9). Im Vergleich der Kohorten, die sich während der Öffnungszeiten der jeweiligen Versorgungsstruktur vorstellten, zeigte sich hinsichtlich der Behandlungsdringlichkeit und des daraus möglicherweise resultierenden diagnostischen Aufwands in dieser Studie kein eindeutiges Muster. Routinedatenanalysen einer anderen Studie weisen jedoch einen höheren Anteil von hoch akuten Patient:innen in Notaufnahmen an Wochentagen im Vergleich zu Wochenenden aus [33], der einen höheren Ressourcenaufwand zumindest in Teilen begründen könnte.

Die Prozessbeobachtungen weisen hinsichtlich der sekundären Versorgungseffizienz in der KV-Notdienstpraxis auf eine konsistentere Berücksichtigung des Behandlungs- und diagnostischen Spektrums der Versorgungsstruktur bei der Steuerung hin. Im Modell der klinikeigenen Ambulanz war an einem Studienzentrum ein direkter Zugang zu Ressourcen der Notaufnahme möglich; die vermehrte Inanspruchnahme diagnostischer und konsiliarischer Ressourcen könnte daher sowohl auf deren unmittelbare Verfügbarkeit als auch auf die in der Notfallversorgung vorherrschende, auf diagnostische Abklärung ausgerichtete Versorgungslogik zurückzuführen sein. Auch der höhere Anteil stationärer Aufnahmen zur Baseline in der klinikeigenen Ambulanz im Vergleich zur KV-Notdienstpraxis (6,2 %, *n *= 9 versus *n *< 5) könnte auf Unterschiede in der Passgenauigkeit der Zuweisung hindeuten.

Die effektivere Versorgung im KV-Kooperationsmodell scheint schließlich eine Versorgungsnachlast für das Gesundheitssystem mit höheren gesellschaftlichen Kosten zu erzeugen, die die ressourcenintensive Versorgung in der klinikeigenen Ambulanz zu kompensieren scheint.

Im 30-Tages-Nachbeobachtungszeitraum lag der Anteil von Notaufnahmewiedervorstellungen bei 10,4 % (*n* = 14) für in der KV-Notdienstpraxis versorgte Patient:innen gegenüber 2,1 % (*n* = 1) für Patient:innen der klinikeigenen Ambulanz; diese waren überwiegend ungeplant und sind trotz geringer Fallzahl als potenziell sicherheitsrelevant einzuordnen. Da die Studienfallzahl für diesen Endpunkt nicht bemessen war, ist dieser Befund explorativ. Die internationale Evidenz zeigt hierzu keine robusten Modellunterschiede [31].

Analog zum höheren Anteil der Wiedervorstellungen in der Notaufnahme im Modell der KV-Notdienstpraxen sind auch die gesellschaftlichen Kosten aufgrund der damit verbundenen stationären Aufnahmen höher.

Aus Sicht der sektoralen Trennung der Versorgungsmandate kann die Versorgungsnachlast grundsätzlich auch als intendierter Effekt interpretiert werden, sofern eine initiale ambulante Akutversorgung erfolgt und eine weiterführende diagnostische Abklärung bzw. Therapie gezielt initiiert wird. Eine solche Weiterleitung wird beispielsweise im Modell der Partnerpraxen pilotiert [34]. Sowohl für dieses Modell als auch für die diskutierte Weiterleitung von Patient:innen aus Krankenhäusern ohne INZ an Standorte mit INZ bleibt unklar, wie ein sicherer Weiterleitungsprozess ausgestaltet werden kann, der die klinische Versorgungskontinuität gewährleistet [13]. Zudem gibt es aktuell zwar Vorschläge [3], aber noch keine Vorgaben zur Ausgestaltung und Ausstattung von Alternativstrukturen sowie zur vorzuhaltenden Fachkompetenz.

#### Hohe Patient:innenzufriedenheit in allen Modellen

In der Studie zeigten sich zwischen den Versorgungsmodellen deskriptiv geringfügige Unterschiede in der Patient:innenzufriedenheit. Angesichts der insgesamt hohen Zufriedenheitswerte lassen sich auf der Mikroebene keine eindeutigen Patient:innenpräferenzen zwischen den untersuchten Modellen ableiten. Vor dem Hintergrund des international beschriebenen Deckeneffekts des eingesetzten Instruments sollte für zukünftige Studien die Verwendung eines alternativen Erhebungsinstruments geprüft werden [35].

Studien weisen darauf hin, dass die subjektiv wahrgenommene Dringlichkeit sowie damit verbundene sozial habitualisierte Erwartungen an eine umfassende Diagnostik in der Notaufnahme die Versorgungserfahrung maßgeblich prägen dürften [36]. Vertiefende Analysen der Patient:innenperspektive auf die Steuerungsmodelle sind sowohl auf Basis teilnehmender Beobachtungen als auch auf Basis von Interviews vorgesehen. Ergänzend wurden Fokusgruppen durchgeführt, um die Versorger:innenperspektive zu erfassen. Für beide Perspektiven sind separate Publikationen geplant.

### Limitationen

#### Übergreifend.

Es wurde kein zeitlicher Verlauf der Notaufnahmeentlastung untersucht; eine Zunahme von hoch akuten Patient:innen bei gleichzeitiger Reduktion wenig akuter Patient:innen in der Notaufnahme bei INZ-Betrieb wurde von anderen Autor:innen berichtet [37]. Die Vergleichbarkeit der Zentren des Studienarms „klinikeigene Ambulanz“ war hinsichtlich bestimmter Struktur- und Prozessmerkmale, wie der Steuerungsmodalität und des Zugriffs auf Notaufnahmeressourcen, eingeschränkt.

#### Primärdatenkohorte.

Eine eingeschränkte Verfügbarkeit der klinikeigenen Ambulanz an einem Standort machte eine Anpassung der Rekrutierung notwendig. Es erfolgte zudem eine eingeschränkte Rekrutierung an Wochenenden. Entsprechend könnte sich die Morbiditätsstruktur der Patient:innenkollektive zwischen den verglichenen Versorgungsstrukturen unterscheiden. Eine an der tatsächlichen Steuerungspraxis orientierte Rekrutierung war aufgrund der nicht standardisierten Steuerung an 2 Standorten erschwert und führte zur Überrekrutierung in der Alternativstruktur (Studienarm 1 und 2).

Aufgrund uneinheitlicher Dokumentation des Ressourcenverbrauchs und von Untersuchungen in den KV-Notdienstpraxen war die Ermittlung der Kosten aus dieser Perspektive nicht möglich. Für den Tag der initialen Notaufnahmevorstellung (t0) konnten lediglich die Kosten der Notaufnahmeversorgung ermittelt werden.

Die Responserate im 30-Tage-Follow-up betrug 47 % und lag damit im erwarteten Bereich. Ein Selektionsbias aufgrund systematischer Unterschiede zwischen Teilnehmenden und Nichtteilnehmenden, die die Versorgungszufriedenheit und die Wiedervorstellung in der Notaufnahme beeinflusst haben könnten, kann jedoch nicht ausgeschlossen werden.

Ärzt:innen im Modell der Standardversorgung nahmen die Einschätzung der Versorgungsebene überwiegend in Kenntnis der Diagnostikergebnisse vor, wodurch ihre Empfehlung zumindest teilweise zugunsten der Versorgungsebene der ambulanten Praxis verschoben sein könnte. Ursprünglich war für diesen Studienarm ein Vergleich mit dem Partnerpraxenmodell vorgesehen, das jedoch in Berlin nicht rechtzeitig zustande kam [10].

#### Routinedatenkohorte.

Teilweise unvollständige Routinedaten erforderten literaturbasierte Annahmen zum Anteil sich selbstständig vorstellender Patient:innen. Für den Anteil der Patient:innen, die durch die KV-Leitstelle eine Zuweisung zur KV-Notdienstpraxis erhielten oder sich dort geplant vorstellen (ohne das Versorgungsziel „Notaufnahme“), liegt kein Proxy vor. Der Entlastungseffekt durch die KV-Notdienstpraxis in der Routinedatenkohorte könnte daher tatsächlich niedriger liegen, da sich Patient:innen auch gezielt für die Versorgung in der KV-Struktur vorstellen könnten.

Die sekundäre Versorgungseffizienz konnte in der Routinedatenkohorte aufgrund unvollständiger Daten nur auf Basis jeweils eines Studienzentrums pro Versorgungsmodell geschätzt werden. Wegen der damit verbundenen möglichen Konfundierung von Zentrumseffekten ist die Primärdatenkohorte für die Interpretation dieses Endpunkts besser geeignet.

## Fazit

In der Zusammenschau der Ergebnisse führen eine eindeutige Trennung der alternativen Versorgungsstruktur von der Notaufnahme am Standort, eine Besetzung durch niedergelassene Ärzt:innen sowie eine Standardisierung des Steuerungsprozesses zu einer höheren Versorgungseffizienz (Anteil alternativ versorgter Fälle). Internationale Literatur bestätigte eine hohe Versorgungseffizienz insbesondere von allgemeinmedizinisch geführten Alternativstrukturen. Dabei zeigt sich ein maximales Entlastungspotenzial von ca. 30 % der Fälle einer Notaufnahmepopulation.

Gleichzeitig weist die KV-Notdienstpraxis jedoch im Vergleich zur klinikeigenen Ambulanz eine höhere Versorgungsnachlast auf, was potenziell auf sicherheitsrelevante Effekte hindeuten könnte. Demgegenüber scheint die umfangreichere Diagnostik in der klinikeigenen Ambulanz erneuten Vorstellungen vorzubeugen. Damit könnten initiale Kostenvorteile der KV-Notdienstpraxis aus Sicht der Notaufnahme durch höhere gesellschaftliche Folgekosten im Nachbeobachtungszeitraum teilweise abgeschwächt oder aufgehoben werden.

Vor diesem Hintergrund erscheint eine gezielte Kombination ausgewählter Struktur- und Prozessmerkmale für die Weiterentwicklung der integrierten Akut- und Notfallversorgung sinnvoll, um eine Entlastung der Notaufnahmen zu erzielen, ohne Versorgungsrisiken für Patient:innen und die Versorgungsnachlast für das Gesundheitssystem zu erhöhen. Insgesamt spricht die Evidenz für eine strukturell und professionell eigenständige, ambulant geführte Versorgungsstruktur, die ein verbindliches Angebot definierter diagnostischer Leistungen anbietet. Weiterleitungen in Partnerpraxen außerhalb des Notaufnahmestandortes zur initialen Versorgung sind derzeit aus Haftungs- und Sicherheitsgründen kritisch zu bewerten, könnten jedoch als sekundärer Behandlungskontakt bei verbindlicher Organisation und Verfügbarkeit in Betracht gezogen werden.

Darüber hinaus unterstreichen die Studienergebnisse die Notwendigkeit validierter und regelhaft angewendeter Steuerungs- und Ersteinschätzungsinstrumente. Die Implementierung alternativer Versorgungsmodelle sollte wissenschaftlich begleitet werden, um Effekte auf der Makro‑, Meso- und Mikroebene der Versorgung systematisch zu erfassen und zu bewerten.

## Supplementary Information

Onlinematerial: eTabellen und eAbbildungen mit ergänzenden Informationen

## Data Availability

Auf berechtigte Anfrage können anonymisierte Daten der Studie ausschnittsweise anderen Arbeitsgruppen für sekundäre Analysen zur Verfügung gestellt werden.

## Footnotes

1 Das den Prozessbeobachtungen zugrunde liegende Beobachtungsprotokoll sowie die typologisch verdichtete Ergebnistabelle befinden sich in E‑Tabelle 1 und E‑Tabelle 10.

2 Primäre Endpunkte werden auf Basis einer unselektierten bzw. selektierten, wenig akuten Studienpopulation als prozentualer Versorgungsanteil mit binomialen 95 %-Konfidenzintervallen ausgegeben. Die *p*-Werte in Tab. 1 beziehen sich auf den Vergleich der primären Versorgungseffizienz zwischen den Studienarmen (Chi^2^-Test).

3 Berechnung basiert auf einem Zentrum.

4 Berechnung basiert auf einem Zentrum.

5 Insgesamt betrug der Anteil direkter stationärer Aufnahmen aus der Wiedervorstellung 21,3 % (*n* = 10, nmiss=2). Für Studienarm 1 erfolgten keine stationären Aufnahmen nach Baselineversorgung in der klinikeigenen Ambulanz, während in Studienarm 2 dieser Anteil 28,6 % (*n* = 4) nach Baselineversorgung in der KV-Notdienstpraxis betrug.

6 Für die Kostenanalyse im Nachbeobachtungszeitraum konnten – anders als bei der Analyse aus Perspektive der jeweiligen Versorgungsstruktur – alle Studienarme und Versorgungsmodelle aufgrund patient:innenberichteter Angaben zur weiteren Inanspruchnahme von Versorgungsleistungen berücksichtig werden.

## References

[1] Krüger D, Möhring F, Steppeler EM et al (2025) Quo vadis: Streaming in emergency and acute care? Results of an online survey on current models of patient streaming and integrated care in emergency departments in Germany. Med Klin Intensivmed Notfmed. 10.1007/s00063-025-01341-110.1007/s00063-025-01341-1 41254189

[2] BMG (2026) Gesetzentwurf der Bundesregierung. Entwurf eines Gesetzes zur Reform der Notfallversorgung. https://www.bundesgesundheitsministerium.de/service/gesetze-und-verordnungen/detail/notfallreform. Zugegriffen: 07.09.2026.

[3] Busch HJ, Hans FP, Pin M et al (2026) Recommendations of the DGIIN and DGINA on the structure and staffing of integrated emergency centres. Med Klin Intensivmed Notfmed 121:169–183. 10.1007/s00063-025-01398-y 41504780

[4] Bessert B, Oltrogge-Abiry JH, Peters PS et al (2023) Synergism of an urgent care walk-in clinic with an emergency department. Dtsch Arztebl Int 120:491–498. 10.3238/arztebl.m2023.0127 PMC10511010 37378594

[5] Seeger I, Rupp P, Naziyok T, Rölker-Denker L, Röhrig R, Hein A (2017) Outpatient care in emergency departments and primary care services: A descriptive analysis of secondary data in a rural hospital. Med Klin Intensivmed Notfmed 112:510–518. 10.1007/s00063-016-0233-1 27837207

[6] Koech L, Ströhl S, Lauerer M et al (2024) Redirection of patients from the emergency department to ambulatory care: a feasibility study. Gesundheitswesen 86:339–345. 10.1055/a-2206-1738 PMC11077551 38354744

[7] Blaschke S, Dormann H, Somasundaram R et al (2025) Structured triage in the emergency department via intelligent assistant service OPTINOFA: results of a multicenter, cluster-randomized and controlled interventional study in Germany. Med Klin Intensivmed Notfmed 120:585–595. 10.1007/s00063-024-01229-6 PMC12504124 39680133

[8] Ansmann L, Baumann W, Gostomzyk J et al (2019) DNVF-Memorandum III: methods for health services research. Part 4 - concept and methods for organizational health services research. Chapter 1 - definition and concept of organizational health services research. Gesundheitswesen 81:e64–e71. 10.1055/a-0862-0527 30952172

[9] Schmiedhofer M, Krüger D, Möckel M, Henschke C, Ansmann L, Slagman A (2026) Organizational context for the work of emergency departments in Germany: status quo, assessment of effects, and need for change for optimized care. Endoscopy 88:148–157. 10.1055/a-2550-9472 40209765

[10] Krüger D, Altendorf M, Holzinger F et al (2025) Patient navigation in German emergency care (NODE): study protocol of a multi-center mixed-methods trial. Front Health Serv 5:1524552. 10.3389/frhs.2025.1524552 PMC12417391 40933718

[11] Legg D, Fischer-Rosinsky A, Holzinger F, Möckel M, Slagman A (2022) Overcoming terminological inconsistency in the study of emergency department attendees who do not require clinically defined emergency care. Eur J Emerg Med 29:395–396. 10.1097/mej.0000000000000981 36094375

[12] Gries A, Schrimpf AM, Dercks NV (2022) Hospital emergency departments: utilization and resource deployment in the hospital as a function of the type of referral. Dtsch Arztebl Int 119:640–646. 10.3238/arztebl.m2022.0276 PMC9764348 35912425

[13] Slagman A, Bremicker A, Möckel M, Eienbröker L, Fischer-Rosinský A, Gries A (2024) Evaluation eines Assistenzsystems zur Patientensteuerung in der Notaufnahme. Dtsch Arztebl International 121:703–709. 10.3238/arztebl.m2024.0191 PMC12005381 39323221

[14] Dinh MM, Berendsen R, Bein KJ et al (2016) Statewide retrospective study of low acuity emergency presentations in New South Wales, Australia: who, what, where and why? BMJ Open 6:e010964. 10.1136/bmjopen-2015-010964 PMC4874101 27165649

[15] Nagree Y, Camarda VJ, Fatovich DM et al (2013) Quantifying the proportion of general practice and low-acuity patients in the emergency department. Med J Aust 198:612–615. 10.5694/mja12.11754 23919709

[16] Stephens AS, Broome RA (2017) Patterns of low acuity patient presentations to emergency departments in New South Wales, Australia. Emerg Med Australas 29:283–290. 10.1111/1742-6723.12767 28320067

[17] O’Dowd TC, McNamara K, Kelly A, O’Kelly F (2006) Out-of-hours co-operatives: general practitioner satisfaction with governance and working arrangements. Eur J Gen Pract 12:15–18. 10.1080/13814780600757195 16945867

[18] Thijssen WA, Wijnen-van Houts M, Koetsenruijter J, Giesen P, Wensing M (2013) The impact on emergency department utilization and patient flows after integrating with a general practitioner cooperative: an observational study. Emerg Med Int 2013:364659. 10.1155/2013/364659 PMC3814098 24282641

[19] van Uden CJ, Crebolder HF (2004) Does setting up out of hours primary care cooperatives outside a hospital reduce demand for emergency care? Emerg Med J 21:722–723. 10.1136/emj.2004.016071 PMC1726502 15496709

[20] Crawford J, Cooper S, Cant R, DeSouza R (2017) The impact of walk-in centres and GP co-operatives on emergency department presentations: a systematic review of the literature. Int Emerg Nurs 34:36–42. 10.1016/j.ienj.2017.04.002 28506567

[21] van Uden CJ, Winkens RA, Wesseling GJ, Crebolder HF, van Schayck CP (2003) Use of out of hours services: a comparison between two organisations. Emerg Med J 20:184–187. 10.1136/emj.20.2.184 PMC1726038 12642541

[22] Doran KM, Colucci AC, Hessler RA et al (2013) An intervention connecting low-acuity emergency department patients with primary care: effect on future primary care linkage. Ann Emerg Med 61:312–321.e317. 10.1016/j.annemergmed.2012.10.021 23261312

[23] van Veelen MJ, van den Brand CL, Reijnen R, van der Linden MC (2016) Effects of a general practitioner cooperative co-located with an emergency department on patient throughput. World J Emerg Med 7:270–273. 10.5847/wjem.j.1920-8642.2016.04.005 PMC5143310 27942343

[24] Dale J, Green J, Reid F, Glucksman E, Higgs R (1995) Primary care in the accident and emergency department: II. Comparison of general practitioners and hospital doctors. Can J Hosp Pharm 311:427–430. 10.1136/bmj.311.7002.427 PMC2550494 7640592

[25] Chmiel C, Wang M, Sidler P, Eichler K, Rosemann T, Senn O (2016) Implementation of a hospital-integrated general practice: a successful way to reduce the burden of inappropriate emergency-department use. Swiss Med Wkly 146:w14284. 10.4414/smw.2016.14284 26963904

[26] Slagman A. Sicherheit der Empfehlung des digitalen Instruments „Strukturierte medizinische Ersteinschätzung Deutschland (SmED) – Kontakt+“ zu Versorgungsebene und Versorgungszeitpunkt beim Einsatz in der Notaufnahme (Poster). DGINA Augsburg2024.

[27] Deutsche Krankenhausgesellschaft (2024ed) Tarif der Deutschen Krankenhausgesellschaft für die Abrechnung erbrachter Leistungen und für die Kostenerstattung vom Arzt an das Krankenhaus zugleich BG‑T vereinbarter Tarif für die Abrechnung mit den gesetzlichen Unfallversicherungsträgern, 43. Aufl. Verlag W. Kohlhammer, Stuttgart

[28] Muntendorf LK, Brettschneider C, Konnopka A, König HH (2024) Updating standardized unit costs from a societal perspective for health economic evaluation. MSK – Muskuloskelettale Physiotherapie 86:389–393. 10.1055/a-2169-1480 PMC11077543 38316405

[29] Rädiker S, Kuckartz U (2019) Analyse qualitativer Daten mit MAXQDA. Springer Nature Link

[30] S J, Nübling R (2002) ZUF‑8. Fragebogen zur Messung der Patientenzufriedenheit. In: Diagnostische Verfahren in der Psychotherapie. Hogrefe, Göttingen, S 392–396

[31] Holzinger F, Schmidt K, Legg D et al (2026) Effectiveness and safety of emergency department-based streaming interventions for low-acuity utilizers: systematic review and meta-analysis. BMC Emerg Med 26:58. 10.1186/s12873-026-01488-w PMC12922365 41714943

[32] Slagman A, Röttger MC, Krug J et al (2026) Evaluation of the TriageClient digital decision support system for the sectoral allocation of emergency patients in an integrated emergency center: a secondary data analysis. Med Klin Intensivmed Notfmed. 10.1007/s00063-026-01455-010.1007/s00063-026-01455-0 42154035

[33] Fischer-Rosinský A, Slagman A, Legg D et al (2024) Frequency, demographics, diagnoses and consultation patterns associated with low-acuity attendances in German emergency departments: a retrospective routine healthcare data analysis from the INDEED project. BMJ Open 14:e084986. 10.1136/bmjopen-2024-084986 PMC11667451 39806647

[34] Oslislo S, Witt K, von Stillfried D et al (2024) Zwischen Vision und Wirklichkeit: Untersuchung zur Machbarkeit der Weiterleitung von weniger dringlichen Hilfesuchenden in die ambulante Versorgung. Notf Rett Med. 10.1007/s10049-024-01347-w

[35] Pedersen H, Havnen A, Brattmyr M, Attkisson CC, Lara-Cabrera ML (2022) A digital Norwegian version of the client satisfaction questionnaire 8: factor validity and internal reliability in outpatient mental health care. BMC Psychiatry 22:671. 10.1186/s12888-022-04281-8 PMC9623922 36316661

[36] Slagman A, Schmiedhofer M, Legg D et al (2025) Reasons for emergency department use of low-acuity attender: results from the prospective, multicentre, cross-sectional EPICS-9/PiNo-Bund study. BMJ Open 15:e090681. 10.1136/bmjopen-2024-090681 PMC11877155 40032406

[37] Hans FP, Röttger MC, Kleinekort J et al (2024) Effekte einer zentralen Ersteinschätzungsstelle mit digital assistierter Triage in einem integrierten Notfallzentrum. Dtsch Arztebl International 121:849–850. 10.3238/arztebl.m2024.0201 PMC12094710 39936316

[38] Ludwig K et al (2018) German Value Set for the EQ-5D-5L. PharmacoEconomics 36:663–674 10.1007/s40273-018-0615-8 PMC5954069 29460066

[39] Brauns H, Scherer S, Steinmann S (2003) The CASMIN educational classification in international comparative research. In: Hoffmeyer-Zlotnik JHP, Wolf C (Hrsg) Advances in cross-national comparison: a European working book for demographic and socio-economic variables. Springer US, Boston, MA, S 221–244

