# Supplementary material for: Efficiency of streaming and integrated care models for low acuity patients in acute and emergency care—results from a prospective cohort study
Source: Bundesgesundheitsblatt Gesundheitsforschung Gesundheitsschutz. 2026 Sep 18;69(10):1183–93. [Article in German] doi: 10.1007/s00103-026-04298-4 (PMC13623996; doi:10.1007/s00103-026-04298-4)
Supplement: Supplementary file 1 [file 103_2026_4298_MOESM1_ESM.pdf]

Onlinematerial: eTabellen und eAbbildungen mit ergänzenden Informationen

Effizienz von Steuerungs- und integrierten Versorgungsmodellen für wenig akute Patient:innen in der Akut- und Notfallversorgung – Ergebnisse einer prospektiven Kohortenstudie

Daniela Krüger[1], Cornelia Wäscher[2], Sylwia Steinke[1], Felix Holzinger[2], Hanna Winkler[3], Martin Möckel[1], Konrad Schmidt[2,4], Anna Slagman[1]

[1]Notfall- und Akutmedizin, Campus Mitte und Virchow, Charité - Universitätsmedizin Berlin, Berlin, Deutschland
[2]Institut für Allgemeinmedizin, Charité - Universitätsmedizin Berlin, Berlin, Deutschland
[3]Institut für Sozialmedizin, Epidemiologie und Gesundheitsökonomie, Charité - Universitätsmedizin Berlin, Berlin, Deutschland
[4]Institut für Allgemeinmedizin, Medizinische Hochschule Brandenburg Theodor Fontane, Brandenburg, Deutschland

Inhalt

METHODIK 3
- Studienarme und beteiligte Studienzentren (eAbbildung 1) 3
- Ärztlicher Bewertungsbogen für die Versorgungsempfehlung in Studienarm 3 (eAbbildung 2) 4
- Beobachtungsprotokoll für die Prozessbeobachtungen (eTabelle 1) 6
- Detaillierte Operationalisierung des primären Endpunktes (eTabelle 2, 3) 8
- Übersicht über die Endpunkte in der NODE-Studie (eTabelle 4) 12
- Annahmen zur Fallzahlplanung (eTabelle 5) 13

ERGEBNISSE 15
- Charakterisierung der Primärdatenkohorte zur Baseline (eTabelle 6, 7) 15
- Charakterisierung der Primärdatenkohorte im Follow-up (eTabelle 8) 18
- Patient:innen-Fluss in der Routinedatenkohorte und Charakterisierung der Patient:innen (eAbbildung 3, eTabelle 9) 20
- Charakterisierung der Steuerungsprozesse und Strukturmerkmale (Studienarm 1, 2; eTabelle 10) 24
- Kosten der Notaufnahmeversorgung (eTabelle 11) 28
- Verfügbarkeit der Steuerungsmodelle (Studienarm 1 und 2; eTabelle 12) 29
- Kosten aus gesellschaftlicher Perspektive (eTabelle 13) 30
- Wartezeit bis zum ersten ärztlichen Kontakt (eAbbildung 4) 31
- Zufriedenheit mit der Versorgung (eAbbildung 5) 32
- Stationäre Aufnahmen im Follow-up (eAbbildung 6) 33

METHODIK

Studienarme und beteiligte Studienzentren (eAbbildung 1)

Die NODE-Studie ist eine prospektive, multizentrische Kohortenstudie mit drei Studienarmen. Die Abbildung stellt die Studienarme mit dem jeweiligen untersuchten Versorgungsmodell sowie die Studienzentren und deren Öffnungszeiten[1] vor.

eAbbildung 1 Studienarme, beteiligte Notaufnahmen und Öffnungszeiten der Alternativstrukturen in der prospektiven, multizentrischen Mixed-Methods-Kohortenstudie NODE

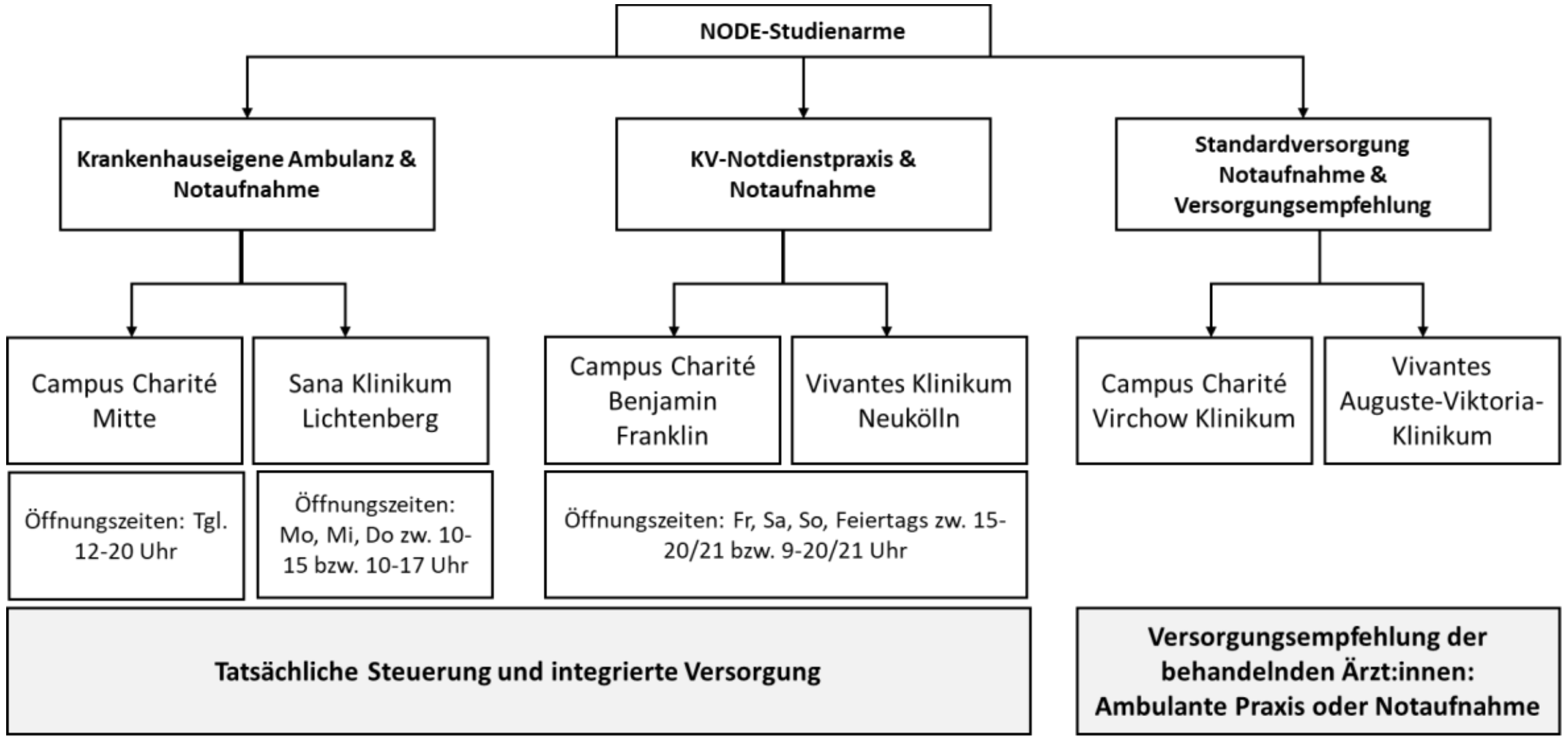

[1]Die Öffnungszeiten der KV-Notdienstpraxen variieren in Berlin saisonal (siehe: https://www.kvberlin.de/fuer-patienten/aerztlicher-bereitschaftsdienst-116117/kv-notdienstpraxen) und sind in folgende Abschnitte unterteilt:

> ***1. April bis 30. September***
> Freitag: 15–20 Uhr; Samstag, Sonntag, Feiertage: 9 –20 Uhr
> ***1. Oktober bis 31. März***
> Freitag: 15–21 Uhr
> **Samstag, Sonntag, Feiertage, 24. und 31. Dezember**
> 9–21 Uh

Ärztlicher Bewertungsbogen für die Versorgungsempfehlung in Studienarm 3 (eAbbildung 2)

Im Studienarm 3 (Standardversorgung) erfolgte auf Grundlage des folgenden Bewertungsbogens eine ärztliche Einschätzung der in die NODE-Studie eingeschlossenen Fälle mit anschließender Versorgungsempfehlung.

eAbbildung 2 Ärztlicher Bewertungsbogen für die Versorgungsempfehlung im Studienarm „Standardversorgung mit ärztlicher Versorgungsempfehlung“ in der prospektiven, multizentrischen Mixed-Methods-Kohortenstudie NODE.

Anmerkung: *In der Studie werden drei Studienarme miteinander verglichen: Studienarm 1 (klinikeigene Ambulanz), Studienarm 2 (KV-Notdienstpraxis) und Studienarm 3 (Standardversorgung mit ärztlicher Einschätzung unter Verwendung eines Bewertungsbogens). Ziel ist es, die Versorgungseffizienz bzw. das Steuerungspotenzial der Modelle zu ermitteln und zu vergleichen. Die Rekrutierung erfolgte vom 01.06.2023 bis zum 31.08.2024 an sechs Studienzentren in Berlin. Der Einsatz des Bewertungsbogens in Studienarm 3 erfolgte an zwei Studienzentren am Tag der Vorstellung in der Notaufnahme. Der Bewertungsbogen wurde entwickelt und validiert im Projekt „SmED-Patient:innensicherheitsstudie“.*[13]

Bewertungsbogen für die Einschätzung zu „Versorgungsebene und –dringlichkeit“

*Bitte möglichst nach Erstkontakt mit dem/der Patient*in ausfüllen*

(Platz für den Patient*innenaufkleber oder SIC)

1. **Aktuelle Uhrzeit:** __ __ : __ __

   Hinweis: Zeitpunkt der Einschätzung durch das ärztliche Personal

2. **Aktuelles Datum:** ___ ___ _________

   Hinweis: Datum der Einschätzung durch das ärztliche Personal

3. **Zeitpunkt des Ausfüllens:**

   Hinweis: Bewertungsbogen wird nach Möglichkeit vor der klinischen Untersuchung ausgefüllt

   ☐ Vor der Untersuchung ☐ Nach der Untersuchung

4. **Aktuelle Mobilität** zum Vorstellungszeitpunkt (Bitte einen auswählen):

   Hinweis: Kann durch Studienassistenz beurteilt werden

   ☐ Gut mobil (fußläufig, eigenständig) ☐ Eingeschränkt mobil (mit Unterstützung) ☐ Nicht ausreichend mobil (z.B. bettlägerig)

5. **Allgemeinzustand** zum Vorstellungszeitpunkt/Symptomatik (Bitte einen auswählen):

   *Hinweis: Zu beurteilen durch den/die behandelnde/n Ärzt*in*

   ☐ Gut bis leicht eingeschränkt (leichte Symptomatik) ☐ Eingeschränkt bis stark eingeschränkt (mittlere bis starke Symptomatik)

6. **Versorgungszeitpunkt** Ihrer Einschätzung nach (Bitte einen auswählen):

   *Hinweis: Zu beurteilen durch den/die behandelnde/n Ärzt*in*

   ☐ Notfall (unverzüglich) ☐ Schnellstmöglich (bis 4 h) ☐ Binnen 24 h ☐ Nicht binnen 24 h erforderlich

7. **Geeigneter Versorgungsort** Ihrer Einschätzung nach (Bitte einen auswählen):

   *Hinweis: Zu beurteilen durch den/die behandelnde/n Ärzt*in; Beurteilung auf Basis Ihrer Einschätzung zu Mobilität, Allgemeinzustand/Symptomatik, Ressourcenbedarf und Versorgungszeitpunkt*

   ☐ Versorgung in der Notaufnahme erforderlich

   Hauptgrund/Hauptgründe für die Zuordnung zur Notaufnahme (*Mehrfachauswahl möglich*):

   ☐ Mobilität ☐ Allgemeinzustand/Symptomatik ☐ Ressourcenbedarf (z.B. Großbild-Diagnostik; Konsile)

   ☐ Versorgungszeitpunkt ☐ Anderes:

   ☐ Versorgung in einer Praxis möglich

Beobachtungsprotokoll für die Prozessbeobachtungen (eTabelle 1)

Zur Charakterisierung der Steuerungsprozesse und der Modellstruktur mittels teilnehmender Beobachtungen in den Studienzentren kamen halbstandardisierte Beobachtungsprotokolle zum Einsatz. Die Prozessbeobachtungen wurden zusätzlich zu teilnehmenden Beobachtungen ganzer Versorgungsverläufe von Patient:innen durchgeführt. Die Ergebnisse dieser Beobachtungen werden anderweitig veröffentlicht.

eTabelle 1 Halbstandardisiertes Beobachtungsprotokoll für die Prozessbeobachtungen in der prospektiven, multizentrischen Mixed-Methods-Kohortenstudie NODE

Anmerkung: In der Studie werden drei Studienarme miteinander verglichen: Studienarm 1 (klinikeigene Ambulanz), Studienarm 2 (KV-Notdienstpraxis) und Studienarm 3 (Standardversorgung mit ärztlicher Einschätzung). Ziel ist es, die Versorgungseffizienz bzw. das Steuerungspotenzial der Modelle zu ermitteln und zu vergleichen. Die Prozessbeobachtungen dienten der Charakterisierung der Modellstrukturen und -prozesse. Die Rekrutierung erfolgte vom 01.06.2023 bis zum 31.08.2024 an sechs Studienzentren in Berlin. Insgesamt wurden n=15 Prozessbeobachtungen durchgeführt.

**VERSORGUNGSSTRUKTUR UND PROZESSBEOBACHTUNG**<br>[Zusammenfassende Beschreibung der Beobachtung, Versorgungsstruktur und deren Umsetzung]	
Studienarm	
Studienzentrum	
Datum	
Beobachtungszeit	
Einwilligung Personal<br><br>a) Pseudonyme<br>b) Form der Einwilligung	
Anzahl<br><br>a) beobachteter Patient*innen in Triage<br>b) vollständig beobachteter Behandlungsverläufe	
Beschreibung der Versorgungsstruktur allgemein	

a) Notaufnahme<br>b) Alternative Versorgung<br><br>(inkl. räumliche Struktur, Fachdisziplinen)	
Heutige Ressourcen- & Personalausstattung sowie Patient*innenaufkommen<br><br>a) Notaufnahme<br>b) Alternative Versorgung	
Patient*innen-Steuerung allgemein<br><br>a) Ablauf<br>b) Zuständigkeit<br>c) Grundlage<br>d) Durchführung<br>e) Sicherheit	
Hinweise aus dem Team zur Wirksamkeit des Modells sowie zu hindernden & fördernden Faktoren für die gemeinsame Versorgung	

Detaillierte Operationalisierung des primären Endpunktes (eTabelle 2, 3)

Die nachfolgende Tabelle bietet eine Übersicht über die Operationalisierung des primären Endpunktes und die zugrunde liegenden Grundgesamtheiten und Datengrundlagen.

eTabelle 2 Übersicht über die Operationalisierung des primären Endpunktes und die zugrunde liegenden Grundgesamtheiten und Datengrundlagen in der prospektiven, multizentrischen Mixed-Methods-Kohortenstudie NODE

Anmerkung: In der Studie werden drei Studienarme miteinander verglichen: Studienarm 1 (klinikeigene Ambulanz), Studienarm 2 (KV-Notdienstpraxis) und Studienarm 3 (Standardversorgung mit ärztlicher Einschätzung). Ziel ist es, die Versorgungseffizienz bzw. das Steuerungspotenzial der Modelle zu ermitteln und zu vergleichen. Die Rekrutierung erfolgte vom 01.06.2023 bis zum 31.08.2024 an sechs Studienzentren in Berlin.

	**Primärer Endpunkt**		**Grundgesamtheit für die Ermittlung der primären Endpunkte**	
	Studienarm 1 und 2: Tatsächliche Steuerung beobachtet	Studienarm 3: Steuerungs-potenzial ermittelt *(Ärztl. Einschätzung)*	Alle Notaufnahme-patient:innen *(unselektiert)*	Wenig akute Patient:innen *(selektiert)*
**Datengrundlage**				
Primärdaten	**x**	**x**	**n.v.**	**x**
Routinedaten	**x**	**n.v.**	**x**	**x**

<u>Legende</u> *n.v. = nicht vorgesehen*

Die folgende Tabelle gibt einen Überblick über die Datenverfügbarkeit sowie die detaillierte Operationalisierung der primären und sekundären Effizienzendpunkte in der Routinedatenkohorte.

eTabelle 3 Detaillierte Operationalisierung des primären Endpunktes für die Routinedatenkohorte in Studienarm 1 und 2 (Steuerungsmodelle) in der prospektiven, multizentrischen Mixed-Methods-Kohortenstudie NODE

Anmerkung: In der Studie werden drei Studienarme miteinander verglichen: Studienarm 1 (klinikeigene Ambulanz), Studienarm 2 (KV-Notdienstpraxis) und Studienarm 3 (Standardversorgung mit ärztlicher Einschätzung). Ziel ist es, die Versorgungseffizienz bzw. das Steuerungspotenzial der Modelle zu ermitteln und zu vergleichen. Die Rekrutierung erfolgte vom 01.06.2023 bis zum 31.08.2024 an sechs Studienzentren in Berlin.

	**Klinikeigene Ambulanz**		**KV-Notdienstpraxis**	
	Studienzentrum A	***Studienzentrum B***	***Studienzentrum C***	***Studienzentrum D***
**Zentrale Dokumentation der Zuweisungsentscheidung im Dokumentationssystem der Notaufnahme zur Berechnung des primären Effizienzendpunktes**	**Nicht vorhanden**	**Vorhanden**	**Vorhanden**	**Nicht vorhanden**
Hinweis zur Berechnung des primären Effizienzendpunktes	Jede Bewegung der Patient:innen wird im Studienzentrum eindeutig durch eine Kombination aus Behandlungsraum und Zeitstempel dokumentiert. Für die Auswertung der initialen Steuerung wurde die erste dokumentierte Behandlung herangezogen. Dabei wurden die Patient:innen anhand der initialen räumlichen Zuordnung der klinikeigenen Ambulanz oder der Notaufnahme (operationalisiert als „alle	Die Auswertung erfolgte auf Basis der zentral dokumentierten initialen Steuerung der Patient:innen. Zur Sicherstellung der Vergleichbarkeit wurde die Grundgesamtheit der Notaufnahme auf Zeiträume mit Öffnung der Alternativstruktur begrenzt. Als Öffnung der Alternativstruktur galt ein Tag der regulären Öffnungszeit, an dem mindestens ein/e Patient:in während der Öffnungszeiten in den Räumlichkeiten der	Die Auswertung erfolgte auf Basis der zentral dokumentierten initialen Steuerung der Patient:innen. Zur Sicherstellung der Vergleichbarkeit wurde die Grundgesamtheit der Notaufnahme auf Zeiträume mit Öffnung der Alternativstruktur begrenzt. Als Öffnung der Alternativstruktur galt ein Tag der regulären Öffnungszeit, an dem mindestens ein/e Patient:in während der Öffnungszeiten in den Räumlichkeiten der KV-	Die Analysen basierten auf zwei unabhängig erhobenen Datensätzen: (1) Aggregierten Behandlungszahlen der im Studienzentrum befindlichen KV-Notdienstpraxis (Verfügbar für Quartal Q3-2023 bis Q3-2024) sowie (2) Behandlungsdaten auf Fallebene der Notaufnahme. Zur Sicherstellung der Vergleichbarkeit wurde die Grundgesamtheit der Notaufnahme auf Zeiträume mit Öffnung der Alternativstruktur begrenzt. Das

	anderen Behandlungsräume“) kategorisiert.<br>Zur Sicherstellung der Vergleichbarkeit wurde die Grundgesamtheit der Notaufnahme auf Zeiträume mit Öffnung der Alternativstruktur begrenzt. Eine Öffnung der Alternativstruktur wurde definiert als ein Tag, an dem mindestens ein/e Patient:in während der Öffnungszeiten in den Räumlichkeiten der klinikeigenen Ambulanz versorgt wurde. Das Versorgungspotenzial wurde ausschließlich für diese Zeiträume bestimmt (siehe sAbbildung 1).	klinikeigenen Ambulanz versorgt wurde. Das Versorgungspotenzial wurde ausschließlich für diese Zeiträume bestimmt (siehe sAbbildung 1).	Notdienstpraxis versorgt wurde. Das Versorgungspotenzial wurde ausschließlich für diese Zeiträume bestimmt (siehe sAbbildung 1).	Versorgungspotenzial wurde ausschließlich für diese Zeiträume bestimmt (siehe sAbbildung 1).<br>Zudem wurden die Grundgesamtheiten von Notaufnahme und KV-Praxis zeitlich harmonisiert. Da Daten aus der KV-Praxis erst ab dem dritten Quartal 2023 vorlagen, wurden für die Notaufnahme ausschließlich Behandlungszahlen ab dem 1. Juli 2023 berücksichtigt. Da die KV-Daten bis einschließlich des dritten Quartals 2024 verfügbar waren, der Rekrutierungszeitraum der Studie jedoch am 31. August 2024 endete, wurden die Fallzahlen für September aus den Behandlungszahlen der KV-Praxis herausgerechnet.
**Zentrale Dokumentation von Versorgungsverläufen zur Berechnung des sekundären Effizienzendpunktes**	**Vorhanden**	**Nicht vorhanden**	**Vorhanden**	**Nicht vorhanden**
Hinweis zur Berechnung des sekundären Effizienzendpunktes	Die Auswertung der Bewegungsvariablen ermöglichte die Operationalisierung von Einweisungen in die Notaufnahme nach	**Keine** Berechnung der sekundären Effizienz: Es lagen keine Bewegungsvariablen bzw. fallübergreifenden Variablen zur Abbildung von Versorgungsverläufen zwischen	Es lagen Bewegungsvariablen mit Zeitstempel zur räumlichen und zeitlichen Zuordnung vor. Diese ermöglichten die Operationalisierung von Einweisungen in die	**Keine** Berechnung der sekundären Effizienz: Es lagen keine Bewegungsvariablen bzw. fallübergreifenden Variablen zur Abbildung von Versorgungsverläufen zwischen

	vorausgegangener Behandlung in der klinikeigenen Ambulanz. Hierzu wurden alle nach der initialen Zuweisung dokumentierten sekundären Behandlungskontakte hinsichtlich ihrer räumlichen Zuordnung analysiert.	der Ambulanz und der Notaufnahme vor.	Notaufnahme nach vorausgegangener Behandlung in der KV-Notdienstpraxis, basierend auf der Analyse aller nach der initialen Zuweisung dokumentierten sekundären Behandlungskontakte hinsichtlich ihrer räumlichen Zuordnung.	der KV-Notdienstpraxis und der Notaufnahme vor.

Hinweis zur Verfügbarkeit und zum Umgang mit Nicht-Verfügbarkeit der alternativen Versorgungsstrukturen in den Studienarmen 1 und 2 (Steuerungsmodelle)

Die alternativen Versorgungsstrukturen wiesen eine unterschiedliche Verfügbarkeit auf, an die die Rekrutierung entsprechend angepasst wurde, wie in der folgenden Tabelle dargestellt.

	**Klinikeigene Ambulanz**		**KV-Notdienstpraxis**	
	Studienzentrum A	***Studienzentrum B***	***Studienzentrum C***	***Studienzentrum D***
**Umgang mit Nicht-Verfügbarkeit der alternativen Versorgungsstruktur**	Aufgrund häufiger Nicht-Verfügbarkeit der klinikeigenen Ambulanz erfolgte die Rekrutierung teilweise auch bei geschlossener Versorgungsstruktur. In diesen Fällen wurden ausschließlich in der Notaufnahme versorgte Patient:innen rekrutiert (n=59). Diese Fälle wurden nicht in die Berechnungen der primären und sekundären Versorgungseffizienz einbezogen und im Artikel entsprechend ausgewiesen.	Während des Rekrutierungszeitraums stand die alternative Versorgungsstruktur grundsätzlich zur Verfügung; temporäre Annahmestopps waren hiervon ausgenommen.	Während des Rekrutierungszeitraums stand die alternative Versorgungsstruktur grundsätzlich zur Verfügung; temporäre Annahmestopps waren hiervon ausgenommen.	Während des Rekrutierungszeitraums stand die alternative Versorgungsstruktur grundsätzlich zur Verfügung; temporäre Annahmestopps waren hiervon ausgenommen.

Übersicht über die Endpunkte in der NODE-Studie (eTabelle 4)

Die folgende Tabelle gibt einen Überblick über die Endpunkte, die Bewertungsdimensionen und die Operationalisierung sowie die geplanten Befragungszeitpunkte und die Stichprobengröße. Das vollständige Studiendesign wurde als Studienprotokoll veröffentlicht [10].

eTabelle 4 Übersicht über die Endpunkte in der prospektiven, multizentrischen Mixed-Methods-Kohortenstudie NODE

Anmerkung: In der Studie werden drei Studienarme miteinander verglichen: Studienarm 1 (klinikeigene Ambulanz), Studienarm 2 (KV-Notdienstpraxis) und Studienarm 3 (Standardversorgung mit ärztlicher Einschätzung). Ziel ist es, die Versorgungseffizienz bzw. das Steuerungspotenzial der Modelle zu ermitteln und zu vergleichen. Die Rekrutierung erfolgte vom 01.06.2023 bis zum 31.08.2024 an sechs Studienzentren in Berlin.

**Endpunkt**	**Bewertungsdimension**	**Operationalisierung bzw. Instrument**	**Zeitpunkt der Messung und geplante Stichprobengröße**
Primärer Endpunkt			
	Primäre Versorgungseffizienz des Versorgungsmodells	Anteil von Patient:innen, welche statt in der Notaufnahme in alternativen Versorgungsstrukturen behandelt werden bzw. nach ärztlicher Einschätzung potentiell in einer alternativen Versorgungsstruktur behandelt werden können.	t0 (n=1.031)
Sekundäre Endpunkte			
	Sekundäre Versorgungseffizienz des Versorgungsmodells	Anteil der Fälle, die tatsächlich in der alternativen Versorgungsstruktur behandelt werden, ohne an die Notaufnahme zurückverwiesen zu werden.	t0 (n=1.031)
	Prozessqualität	Prozesszeiten (z.B. Behandlungsdauer, Zeit bis zu den diagnostischen Maßnahmen), Häufigkeit diagnostischer Verfahren	t0 (n=1.031) & t1 (n=344)
	Patient:innensicherheit	Unerwünschte Ereignisse:<br>Mortalität, ungeplante (erneute) Einweisung auf die Intensivstation, ungeplante stationäre Aufnahme, ungeplanter erneuter Besuch der Notaufnahme innerhalb von 30 Tagen.	t0 (n=1.031) & t1 (n=344)
	Patient:innenzufriedenheit	ZUF-8 [30]	t1 (n=344)
	Gesundheitsbezogene Lebensqualität	EQ-5D-5L [38]	t1 (n=344)
	Kosten und Kostenwirksamkeit	Kosten berechnet auf der Grundlage der Inanspruchnahme von: Krankenhausaufenthalten, ambulanten Behandlungen, stationären Aufenthalten, Medikamenten, Hilfsmitteln, diagnostischen und therapeutischen Maßnahmen, Gesamtkosten in Bezug auf Patient:innensicherheit.	t0 (n=1.031) & t1 (n=344)
	Weitere klinische Endpunkte (Analyse abhängig von einer ausreichenden Anzahl von Fällen)	Dauer des stationären Aufenthalts, Häufigkeit des Aufenthalts auf der Intensivstation, Dauer des Aufenthalts auf der Intensivstation zu t0 und t1, falls zutreffend.	t0 (n=1,031) & t1 (n=344)

Legende*:* EQ-5D-5L = 5-stufige EQ-5D-Version des Fragebogens der EuroQoL-Gruppe. ZUF-8 = „Fragebogen zur Messung der Patientenzufriedenheit“. T0 = Ausgangswert, t1 = Nachuntersuchung.

Annahmen zur Fallzahlplanung (eTabelle 5)

Grundgesamtheiten und Versorgungsanteile

Auf Basis der wissenschaftlichen Literatur wurde von unterschiedlichen Versorgungsanteilen ausgegangen, welche in Bezug zur Studienpopulation als Grundgesamtheit gesetzt werden (fußläufig, selbständig vorstellig werdende Patient:innen) [14–26]. Zusätzlich wurden diese Anteile relativ zu dem in der

Literatur berichteten Anteil von 45 % selbstständig in der Notaufnahme vorstellender Patient:innen gesetzt [12], um Vergleiche mit weiteren, in der Literatur verwendeten unselektierten Grundgesamtheiten zu ermöglichen.

Fallzahlschätzung

Für eine zuverlässige Schätzung des Anteils des primären Endpunkts wird eine Präzision von 5% auf Basis des binomialen 95%-Konfidenzintervalls angenommen. Daraus ergaben sich folgende Fallzahlen zur Schätzung der angenommenen Versorgungsanteile des primären Endpunkts in den verschiedenen Studienarmen:

Arm 1 – Klinikeigene Ambulanz: geschätzter Anteil 30 % – n=323

Arm 2 – KV-Notdienstpraxis: geschätzter Anteil 50 % – n=385

Arm 3 – Standardversorgung mit ärztlicher Bewertung: geschätzter Anteil 70 % – n=323

Die Gesamtstichprobengröße beträgt n=1.031. Angenommen war, dass etwa ein Drittel der rekrutierten Patient:innen (n=344) am 30 Tage-Follow-up teilnimmt.

eTabelle 5 Grundgesamtheiten und darauf bezogene Annahmen zu Versorgungsanteilen bzw. zum ärztlich geschätzten Steuerungspotenzial in der prospektiven, multizentrischen Mixed-Methods-Kohortenstudie NODE

Anmerkung: In der Studie werden drei Studienarme miteinander verglichen: Studienarm 1 (klinikeigene Ambulanz), Studienarm 2 (KV-Notdienstpraxis) und Studienarm 3 (Standardversorgung mit ärztlicher Einschätzung). Ziel ist es, die Versorgungseffizienz bzw. das Steuerungspotenzial der Modelle zu ermitteln und zu vergleichen. Die Rekrutierung erfolgte vom 01.06.2023 bis zum 31.08.2024 an sechs Studienzentren in Berlin.

	**Klinikeigene Ambulanz**	**KV-Notdienstpraxis**	**Standardversorgung mit ärztlicher Einschätzung der Versorgungsebene**
Geschätzter Anteil für wenig akute Patient:innen (*selektierte Kohorte*)	30 %	50 %	70 %
Geschätzter Anteil für alle Notaufnahmepatient:innen (*unselektierte Kohorte*)	14 %	23 %	-

ERGEBNISSE

Charakterisierung der Primärdatenkohorte zur Baseline (eTabelle 6, 7)

Die folgende Tabelle stellt die Baseline-Charakteristika der Primärdatenkohorte am Vorstellungstag in der Notaufnahme für die Gesamtkohorte sowie stratifiziert nach Studienarmen dar. Ergänzend wird ein tabellarischer Vergleich der eingeschlossenen Studienkohorte und der ausgeschlossenen Patient:innen anhand soziodemografischer Kernmerkmale gezeigt.

eTabelle 6 Soziodemografische Baseline-Charakteristika und Triagekategorie der Primärdatenkohorte der prospektiven, multizentrischen Mixed-Methods-Kohortenstudie NODE in der Notfall- und Akutversorgung in Berlin

Anmerkung: Die Baseline-Charakteristika sind für die Gesamtkohorte sowie stratifiziert nach Studienarmen dargestellt (Studienarm 1: klinikeigene Ambulanz; Studienarm 2: KV-Notdienstpraxis; Studienarm 3: Standardversorgung mit ärztlicher Einschätzung). Die Fallzahlen basieren auf der Baseline-Befragung am Tag der Vorstellung. Die Rekrutierung der Primärdatenkohorte erfolgte vom 01.06.2023 bis zum 31.08.2024 an sechs Studienzentren.

		**Gesamtkohorte**		**Klinikeigene Ambulanz**		**KV-Notdienstpraxis**		**Standardversorgung**	
		M	SD	M	SD	M	SD	M	SD
**Alter**		39	14	38	13	40	15	40	15
		N	%	N	%	N	%	N	%
**Geschlecht**	Männlich	499	46%	169	49%	174	42%	156	49%
	Weiblich	573	53%	175	51%	235	57%	163	51%
	Divers	8	1%	>5	-	>5	-	>5	-
	Total	*1082*	*100%*	*346*	*100%*	*414*	*100%*	*320*	*100%*
**Migrationskontext**	Kein Migrationskontext	652	63%	185	56%	263	65%	204	67%
	Migration der ersten Generation	256	25%	118	36%	78	19%	60	20%
	Migration der zweiten Generation	132	13%	26	8%	64	16%	42	14%
	Total	*1040*	*100%*	*329*	*100%*	*405*	*100%*	*306*	*100%*
**Bildungsstatus (CASMIN)**	Niedrig	73	8%	24	8%	26	8%	23	9%
	Mittel	408	45%	130	43%	158	46%	120	46%

	Hoch	427	47%	151	50%	159	46%	117	45%
	Total	*908*	*100%*	*305*	*100%*	*343*	*100%*	*260*	*100%*
	Total	*209*	*100%*	*71*	*100%*	*83*	*100%*	*55*	*100%*
**Wohnort**	Berlin	954	90%	294	87%	382	93%	278	89%
	Brandenburg	42	4%	13	4%	11	3%	18	6%
	Weder noch (Besuch)	64	6%	31	9%	18	4%	15	5%
	Total	*1060*	*100%*	*338*	*100%*	*411*	*100%*	*311*	*100%*
**Triagekategorie**	1. Sofort – rot	-	-	-	-	-	-	-	-
	2. Sehr dringend – orange	21	2%	-	-	12	3%	9	3%
	3. Dringend – gelb	299	28%	71	21%	77	20%	151	46%
	4. Standard – grün	644	61%	261	76%	231	60%	152	47%
	5. Nicht dringend – blau	93	9%	12	3%	67	17%	14	4%
	Total	1057	100%	344	100%	387	100%	326	100%

Legende: *M=Mittelwert; SD=Standardabweichung; CASMIN=Comparative Analysis of Social Mobility in Industrial Nations [39];<5 = Fallzahl unter 5 Angaben nicht berichtet*

eTabelle 7 Baseline-Charakteristika der Primärdatenkohorte der prospektiven, multizentrischen Mixed-Methods-Kohortenstudie NODE sowie der ausgeschlossenen Patient:innen

Anmerkung: Die Baseline-Charakteristika sind stratifiziert nach Studienarmen dargestellt (Studienarm 1: klinikeigene Ambulanz; Studienarm 2: KV-Notdienstpraxis; Studienarm 3: Standardversorgung mit ärztlicher Einschätzung). Die Fallzahlen basieren auf der Baseline-Befragung am Tag der Vorstellung. Die Rekrutierung der Primärdatenkohorte erfolgte vom 01.06.2023 bis zum 31.08.2024 an sechs Studienzentren.

		**Klinikeigene Ambulanz**		**KV-Notdienstpraxis**		**Standardversorgung**	
			**Studienpopulation**				
		M	SD	M	SD	M	SD
**Alter**		38	13	40	15	40	15
		N	%	N	%	N	%
**Geschlecht**	Männlich	169	49%	174	42%	156	49%
	Weiblich	175	51%	235	57%	163	51%

	Divers	>5	-	>5	-	>5	-
	Total	*346*	*100%*	*414*	*100%*	*320*	*100%*
**Staatsangehörigkeit**	Deutsch	238	68%	354	85%	265	81%
	Nicht-Deutsch	114	32%	63	15%	62	19%
	Total	*352*	*100%*	*417*	*100%*	*327*	*100%*
**Ausgeschlossene Patient:innen**							
		M	SD	M	SD	M	SD
**Alter**		42	14	47	19	40	14
		N	%	N	%	N	%
**Geschlecht**	Männlich	166	46%	111	46%	250	47%
	Weiblich	192	53%	130	54%	285	53%
	Divers	>5	-	-	-	-	-
	Total	*359*	*100%*	*241*	*100%*	*535*	*100%*
**Staatsangehörigkeit**	Deutsch	224	66%	191	81%	350	69%
	Nicht-Deutsch	117	34%	46	19%	158	31%
	Total	*341*	*100%*	*237*	*100%*	*508*	*100%*

Legende: *M=Mittelwert; SD=Standardabweichung; N=Fallzahl; <5 = Fallzahl unter 5 Angaben nicht berichtet*

Charakterisierung der Primärdatenkohorte im Follow-up (eTabelle 8)

Die folgende Tabelle stellt die Baseline-Charakteristika der Primärdatenkohorte im 30-Tages-Nachbeobachtungszeitraum für die Gesamtkohorte sowie stratifiziert nach Studienarmen dar.

eTabelle 8 Soziodemografische Baseline-Charakteristika und Triagekategorie der Primärdatenkohorte im Follow-up in der prospektive, multizentrischen Mixed-Methods-Kohortenstudie NODE

Anmerkung: Die Baseline-Charakteristika sind für die Gesamtkohorte sowie stratifiziert nach Studienarmen dargestellt (Studienarm 1: klinikeigene Ambulanz; Studienarm 2: KV-Notdienstpraxis; Studienarm 3: Standardversorgung mit ärztlicher Einschätzung). Die Fallzahlen basieren auf der Follow-up-Befragung, 30 Tage nach dem Tag der Notaufnahmevorstellung. Die Rekrutierung der Primärdatenkohorte erfolgte vom 01.06.2023 bis zum 31.08.2024 an sechs Studienzentren.

		Gesamtkohorte		Klinikeigene Ambulanz		KV-Notdienstpraxis		Standardversorgung	
		M	SD	M	SD	M	SD	M	SD
**Alter**		40.7	14.35	40.45	13.18	40.66	14.63	41.05	15.20
		N	%	N	%	N	%	N	%
**Geschlecht**	Männlich	225	44%	71	45%	79	39%	75	49%
	Weiblich	281	55%	84	54%	119	59%	78	51%
	Divers	5	1%	<5	-	<5	-	<5	-
	Total	*511*	*100%*	*157*	*100%*	*201*	*100%*	*153*	*100%*
**Migrationskontext**	Kein Migrationskontext	345	69%	94	61%	136	69%	115	76%
	Migration der ersten Generation	109	22%	51	33%	38	19%	20	13%
	Migration der zweiten Generation	50	10%	9	6%	24	12%	17	11%
	Total	*504*	*100%*	*154*	*100%*	*198*	*100%*	*152*	*100%*
**Bildungsstatus (CASMIN)**	Niedrig	27	6%	10	7%	8	5%	9	7%
	Mittel	187	42%	57	39%	71	41%	59	46%
	Hoch	232	52%	78	54%	93	54%	61	47%
	Total	*446*	*100%*	*145*	*100%*	*172*	*100%*	*129*	*100%*
**Wohnort**	Berlin	460	91%	136	87%	188	94%	136	90%
	Brandenburg	20	4%	6	4%	<5	-	10	7%
	Weder noch (Besuch)	28	6%	14	9%	9	5%	5	3%
	Total	*508*	*100%*	*156*	*100%*	*201*	*100%*	*151*	*100%*

**Triagekategorie**	1. (SOFORT - rot)	<5	-	<5	-	<5	-	<5	-
	2. (SEHR DRINGEND - orange)	15	3%	<5	-	8	4%	7	5%
	3. (DRINGEND - gelb)	153	31%	36	24%	39	21%	78	51%
	4. (NORMAL - grün)	279	57%	108	72%	108	58%	63	41%
	5. (NICHT DRINGEND - blau)	42	9%	7	5%	30	16%	5	3%
	Gesamt	*489*	*100%*	*151*	*100%*	*185*	*100%*	*153*	*100%*

Legende: *M=Mittelwert; SD=Standardabweichung; N=Fallzahl; %=Spaltenprozente; CASMIN=Comparative Analysis of Social Mobility in Industrial Nations [39];<5 = Fallzahl unter 5 Angaben nicht berichtet*

Patient:innen-Fluss in der Routinedatenkohorte und Charakterisierung der Patient:innen (eAbbildung 3, eTabelle 9)

Die Abbildung zeigt den Patient:innen-Fluss der Routinedatenkohorte der NODE-Studie für die Studienarme 1 und 2 mit tatsächlicher Patient:innensteuerung. Die nachfolgende Tabelle zeigt eine Basis-Charakterisierung der Routinedatenkohorte, stratifiziert nach Vorstellungen innerhalb und außerhalb der Öffnungszeiten der Alternativstruktur. Aus Gründen der Pseudonymisierung ist keine stratifizierte Charakterisierung nach Studienarm und Versorgungssetting möglich, da für einen Studienstandort im Studienarm 2 keine Daten auf Fallebene zur Basis-Charakterisierung vorliegen.

eAbbildung 3 Patient:innen-Fluss in der Routinedatenkohorte der prospektiven, multizentrischen Mixed-Methods-Kohortenstudie NODE (nur Steuerungsmodelle = Studienarm 1: klinikeigene Ambulanz; Studienarm 2: KV-Notdienstpraxis)

Anmerkung: Es werden die Fälle dargestellt, die in die Berechnung des primären Endpunktes eingeflossen sind bzw. von den Berechnungen ausgeschlossen wurden. Die Fallzahlen basieren auf der Routinedatenextraktion der kooperierenden Notaufnahmen mit tatsächlicher Patient:innen-Steuerung (n=4). Es wurden alle volljährigen Patient:innen der Notaufnahme eingeschlossen, die sich im Rekrutierungszeitraum vorstellten: 01.06.2023 bis zum 31.08.2024. In einem Studienzentrum des Studienarms der KV-Notdienstpraxen standen Notaufnahmedaten auf Fallebene und aggregierte Daten der KV-Notdienstpraxis zur Verfügung; letztere wurden in das Flow Chart integriert.

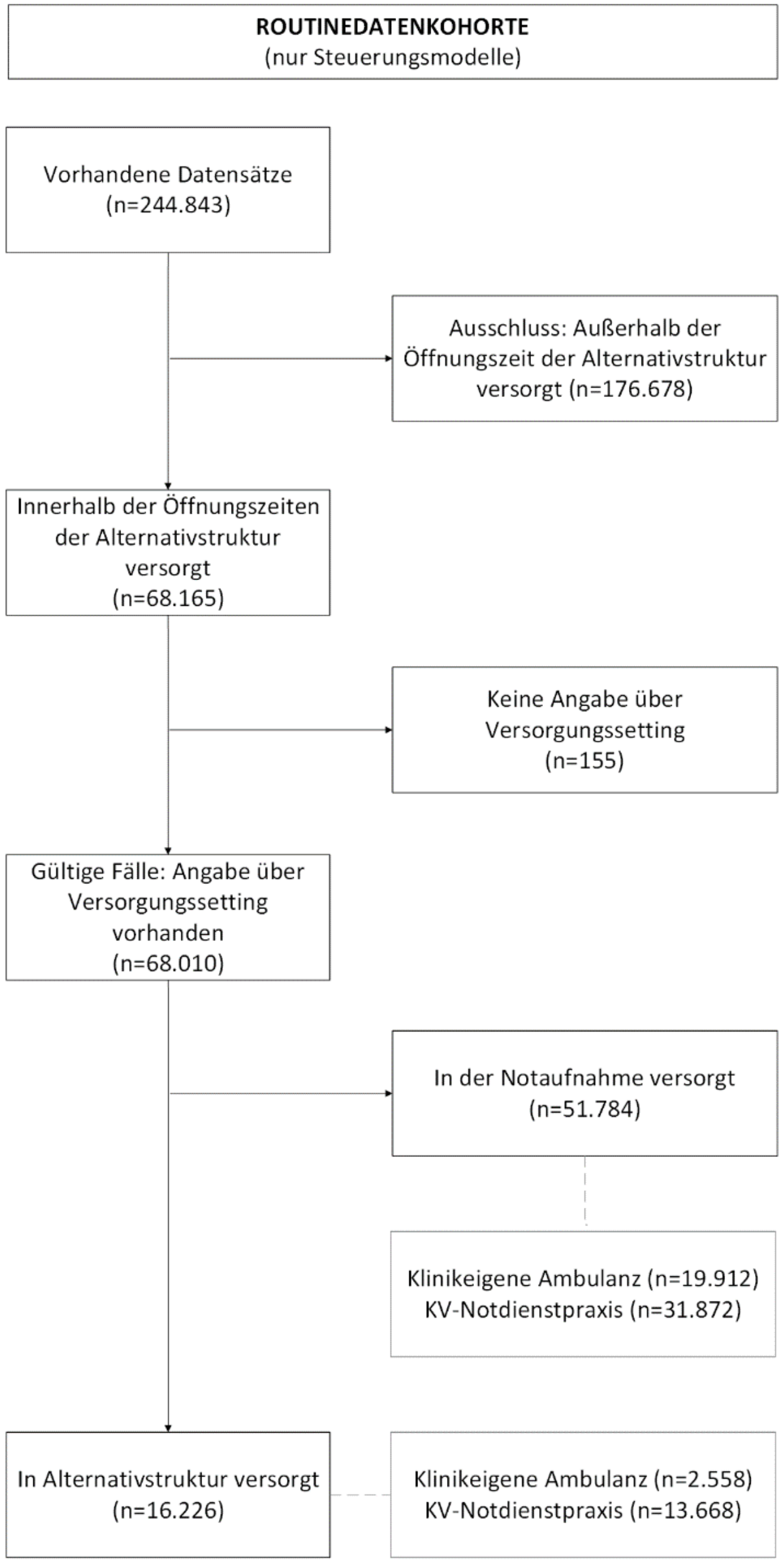
ROUTINEDATENKOHORTE
(nur Steuerungsmodelle)
Vorhandene Datensätze
(n=244.843)
Ausschluss: Außerhalb der Öffnungszeit der Alternativstruktur versorgt (n=176.678)
Innerhalb der Öffnungszeiten der Alternativstruktur versorgt
(n=68.165)
Keine Angabe über Versorgungssetting
(n=155)
Gültige Fälle: Angabe über Versorgungssetting vorhanden
(n=68.010)
In der Notaufnahme versorgt
(n=51.784)
Klinikeigene Ambulanz (n=19.912)
KV-Notdienstpraxis (n=31.872)
In Alternativstruktur versorgt
(n=16.226)
Klinikeigene Ambulanz (n=2.558)
KV-Notdienstpraxis (n=13.668)

eTabelle 9 Routinedatenpopulation: Gegenüberstellung aller volljährigen, zentral in der Notaufnahme registrierten Patient:innen, die sich außerhalb oder innerhalb der regulären ambulanten Sprechzeiten (alle Wochentage) vorgestellt haben in der prospektiven, multizentrischen Mixed-Methods-Kohortenstudie NODE

Anmerkung: Die Fallzahlen basieren auf der Routinedatenextraktion der kooperierenden Notaufnahmen mit tatsächlicher Patient:innensteuerung (n=4) in Berlin. Eingeschlossen wurden alle volljährigen Patient:innen, die sich im Rekrutierungszeitraum vom 01.06.2023 bis zum 31.08.2024 in der Notaufnahme vorstellten. In einem Studienzentrum des Studienarms der KV-Notdienstpraxis lagen Notaufnahmedaten auf Fallebene sowie ausschließlich aggregierte Daten der KV-Notdienstpraxis vor. Die Darstellung erfolgt unter Ausschluss der Patient:innen der KV-Notdienstpraxis dieses Studienzentrums, für die keine soziodemografischen Basismerkmale auf Fallebene verfügbar waren. Eine individuelle Ausweisung dieser Fallzahl ist aus Gründen der Pseudonymisierung der Studienzentren nicht möglich.

		**Studienarm 1: Klinikeigene Ambulanz**								**Studienarm 2: KV-Notdienstpraxis**							
		**Öffnungszeiten der Alternativstruktur**								**Öffnungszeiten der Alternativstruktur**							
		**Vorstellung Außerhalb**				**Vorstellung Innerhalb**				**Vorstellung Außerhalb**				**Vorstellung Innerhalb**			
		M	SD	N	%	M	SD	N	%	M	SD	N	%	M	SD	N	%
Alter		48	20			49	21			56	22			52	22		
Geschlecht	männlich			41.122	49,0%			10.778	48,0%			47.264	51,0%			17.872	47,3%
	weiblich			42.807	51,0%			11.690	52,0%			45.457	49,0%			19.884	52,7%
	divers			<5	-			<5	-			-	-			<5	-
	Gesamt			83.933	100,0%			22.470	100,0%			92.721	100,0%			37.757	100,0%
Triagekategorie	Direkter Arztkontakt			-	-			-	-			-	-			-	-
	SOFORT - rot			310	0,4%			64	0,3%			2.097	2,3%			558	1,5%
	SEHR DRINGEND - orange			5.313	6,4%			1.333	6,1%			14.851	16,2%			4.888	13,0%
	DRINGEND - gelb			35.389	42,9%			9.247	42,3%			35.575	38,8%			11.786	31,4%
	NORMAL - grün			39.233	47,5%			10.440	47,8%			36.257	39,5%			16.144	43,0%
	NICHT DRINGEND - blau			2.277	2,8%			755	3,5%			2.987	3,3%			4.193	11,2%
	Gesamt			82.522	100,0%			21.839	100,0%			91.767	100,0%			3.7569	100,0%
Wochentag der Vorstellung	Sonntag			12.324	14,7%			1.271	5,7%			5.390	5,8%			13.368	35,4%
	Montag			10.758	12,8%			5.514	24,5%			17.748	19,1%			1.000	2,6%
	Dienstag			12.863	15,3%			2.101	9,4%			16.923	18,2%			512	1,4%

	Mittwoch		10.192	12,1%			5.282	23,5%			17.205	18,6%			174	0,5%
	Donnerstag		10.615	12,6%			4.769	21,2%			17.270	18,6%			243	0,6%
	Freitag		13.614	16,2%			2.326	10,4%			12.634	13,6%			7.156	18,9%
	Samstag		13.573	16,2%			1.207	5,4%			5.569	6,0%			15.318	40,6%
	Gesamt		83.939	100,0%			22.470	100,0%			92.739	100,0%			37.771	100,0%
Uhrzeit der Vorstellung	00:00–08:00		13.966	16,6%			<5	-			16.310	17,6%			-	-
	08:01–12:00		22.229	26,5%			4.067	18,1%			22.381	24,1%			8.854	23,4%
	12:01–16:00		15.468	18,4%			12.814	57,0%			22.415	24,2%			13.028	34,5%
	16:01–20:00		16.582	19,8%			5.587	24,9%			14.573	15,7%			13.532	35,8%
	20:01–23:59		15.694	18,7%			-	-			17.060	18,4%			2.357	6,2%
	Gesamt		83.939	100,0%			22.470	100,0%			92.739	100,0%			37.771	100,0%

Legende: *M=Mittelwert; SD=Standardabweichung; N=Fallzahl; %=Spaltenprozente; <5 = Fallzahl unter 5 Angaben nicht berichtet; -=keine Beobachtung*

Charakterisierung der Steuerungsprozesse und Strukturmerkmale (Studienarm 1, 2; eTabelle 10)

eTabelle 10 Typologisch verdichtete Ergebnismatrix[1] zu Steuerungs- und Strukturmerkmalen aus der qualitativen Prozessbeobachtung in der prospektiven, multizentrischen Mixed-Methods-Kohortenstudie NODE

Anmerkung: In der Studie werden drei Studienarme miteinander verglichen: Studienarm 1 (klinikeigene Ambulanz), Studienarm 2 (KV-Notdienstpraxis) und Studienarm 3 (Standardversorgung mit ärztlicher Einschätzung). Ziel ist es, die Versorgungseffizienz bzw. das Steuerungspotenzial der Modelle zu ermitteln und zu vergleichen. Die Rekrutierung erfolgte vom 01.06.2023 bis zum 31.08.2024 an sechs Studienzentren in Berlin.

[1]Insgesamt wurden n = 15 Prozessbeobachtungen an den sechs Studienzentren in allen Studienarmen durchgeführt; die hier berichteten Ergebnisse entsprechen einer Ergebnisauswahl und beziehen sich auf die Prozessbeobachtungen in den Modellen der tatsächlichen Steuerung (Studienarm 1,2; n=11).

	**Klinikeigene Ambulanz**		**KV-Notdienstpraxis**	
	**Standort 1**	**Standort 2**	**Standort 3**	**Standort 4**
**STRUKTURMERMALE**				
Räumlich	Behandlung in separatem Raum innerhalb der Notaufnahme	Behandlung in separatem Raum im Gebäudekomplex der Notaufnahme	Behandlung in separatem Raum innerhalb der Notaufnahme	Behandlung im Gebäudekomplex der Notaufnahme
Personell	Klinikeigenes Personal der Notaufnahme: Wechselnde/r Ärzt:in, feste Pflegekraft	Klinikeigenes Personal: Feste/r Ärzt:in der Allgemeinmedizin und Pflegekraft	Wechselnde Ärzt:innen der KV (verschiedene Fachrichtungen); wechselnde klinikeigene Pflegekraft	Wechselnde Ärzt:innen der KV (verschiedene Fachrichtungen); wechselnde klinikeigene Pflegekraft
Ausstattung	Sonografiegerät (wechselnde Kompetenz zur Diagnostik), EKG; Zugriff auf Notaufnahmeressourcen (Konsil, Labor, Röntgen, CT) möglich	Sonografiegerät (ohne Kompetenz zur Diagnostik), EKG; Labor wie KV-Praxis (nicht taggleich)	Sonografie- und EKG in der Notaufnahme ausleihbar (wechselnde Kompetenz zur Diagnostik); Röntgen und Labor über die Notaufnahme bestellbar	Sonografie- und EKG in der Notaufnahme ausleihbar (wechselnde Kompetenz zur Diagnostik); Labor über die Notaufnahme bestellbar
Öffnungszeiten	Innerhalb und außerhalb der regulären Sprechstundenzeiten: Täglich; 12-20 Uhr	Innerhalb der regulären Sprechstundenzeiten: Mo, Mi, Do zw. 10-15 bzw. 10-17 Uhr	Außerhalb der regulären Sprechstundenzeiten: Fr, Sa, So zw. 15-20/21 bzw. 9-20/21 Uhr	Außerhalb der regulären Sprechstundenzeiten: Fr, Sa, So zw. 15-20/21 bzw. 9-20/21 Uhr
Sektorale Abgrenzung	Struktur in Notaufnahme integriert; direkter Zugriff auf Notaufnahmeressourcen möglich.	Affirmativ: kein Zugriff auf Notaufnahmeressourcen.	Eher Affirmativ: optionale Ausleihe eines Sonografiegerätes möglich sowie Zugriff auf Röntgen- und Labordiagnostik.	Eher Affirmativ: abgesehen von der optionalen Ausleihe eines Sonografiegeräts aus der Notaufnahme und der Möglichkeit zur Labordiagnostik besteht kein Zugriff auf Ressourcen der Notaufnahme.
**PROZESSMERKMALE**				
**Umsetzung allgemein**	Dezentral	Zentral	Zentral	Dezentral

Kurzbeschreibung	Zuweisung vor, während und nach der Triage beobachtet (vgl. sAbbildung 4)	Zuweisung in der Triage (vgl. sAbbildung 4)	Zuweisung in der Triage (vgl. sAbbildung 4)	Zuweisung vor der Triage (durch vorgelagerte Sichtung) sowie durch Selbststeuerung der Patient:innen (vgl. sAbbildung 4)
**Grundlage**	Nicht formalisiert	SOP	Internes Schema	Nicht formalisiert
Kurzbeschreibung	Steuerung in der Regel nach der Triage; Initiierung durch Mitarbeitende (Ärzt:in/Pflegekraft) der ambulanten Versorgungsstruktur bzw. in Absprache mit der Triagekraft oder dem ZNA-Team; kein eigenständiges Aufsuchen der Ambulanz durch Patient:innen möglich.	Steuerung erfolgte in der Triage durch Triagekraft auf Basis einer im Notaufnahmesystem hinterlegten, internen SOP: auf Grundlage der Triage (MTS) und des Behandlungsspektrums der/des ambulanten Ärzt:in; kein eigenständiges Aufsuchen der Ambulanz durch Patient:innen beobachtet, durch räumliche Trennung aber möglich.	Steuerung erfolgte in der Triage durch Triagekraft auf Basis eines internen Schemas: basierend auf Leitsymptomatik und in Abstimmung mit dem Behandlungsspektrum der ambulanten Vertragsärzt:innen; kein eigenständiges Aufsuchen der KV-Praxis durch Patient:innen möglich.	Patient:innen konnten sich selbstständig für die Alternativstruktur vorstellen; Patient:innen, die sich gezielt für die Notaufnahme vorstellten, wurden nach leitsymptomorientierter Einschätzung und unter Berücksichtigung des Behandlungsspektrums der Vertragsärzt:innen weitergeleitet; eigenständiges Aufsuchen der KV-Praxis durch Patient:innen möglich.
**Stabilität der Struktur**	Sehr niedrig	Niedrig	Hoch	Hoch
Kurzbeschreibung	Wiederholte Schließung der Struktur kurz nach Öffnung aufgrund eines erhöhten personellen Mehrbedarfs in der Notaufnahme.	Wiederholte Schließung bei Krankheit/Urlaub der Behandler:in der Alternativstruktur.	Hohe Verlässlichkeit bei der Verfügbarkeit der Alternativstruktur; temporäre Abmeldungen der Praxis innerhalb einer Schicht in Abhängigkeit vom Workflow beobachtet.	Hohe Verlässlichkeit bei der Verfügbarkeit der Alternativstruktur; temporäre Abmeldungen der Praxis innerhalb einer Schicht in Abhängigkeit vom Workflow beobachtet.
**Versorgungslogik**	Notfallmedizinisch	Ambulant-hausärztlich	Ambulant	Ambulant
Kurzbeschreibung	Fallabschließende notfallmedizinische Versorgung mit Ausschlussdiagnostik und Diagnose.	Fokus liegt auf einer allgemeinmedizinisch, hausärztlichen Versorgung, inkl. Aufbau eines Patient:innenstamms.	Fokus liegt auf einer ambulant-akutmedizinischen Versorgung in Abhängigkeit des Behandlungsspektrums der KV-Ärzt:innen, ggf. mit Empfehlung zur Weiterversorgung.	Fokus liegt auf einer ambulant-akutmedizinischen Versorgung in Abhängigkeit des Behandlungsspektrums der KV-Ärzt:innen, ggf. mit Empfehlung zur Weiterversorgung.
**Zielpopulation**	Situativ	Eher enger definiert	Eher enger definiert	Situativ
Kurzbeschreibung	Fälle werden im direkten kommunikativen Austausch zwischen Behandler:in und Pflegekraft individuell ausgewählt. Patient:innen mit Einweisung oder Rettungsdiensttransport können	Empfehlung erfolgt auf Basis der SOP durch ein algorithmengestütztes Triageprogramm; situativ mit Behandler:in bzw. Pflegekraft der Alternativstruktur abgestimmt; Behandlungsspektrum der/des	Zuweisung erfolgt auf Basis eines internen Schemas sowie in Abstimmung mit Behandlungsspektrum der/des KV-Ärzt:in erfolgt; keine Patient:innen mit Einweisung oder Rettungsdiensttransport.	Zuweisung erfolgt auf Basis der berichteten Beschwerde (Ersteindruck) und des Behandlungsspektrums der/des KV-Ärzt:in; keine Patient:innen mit Einweisung oder Rettungsdiensttransport.

	in Alternativstruktur behandelt werden.	Ärzt:in bekannt. Keine Patient:innen mit Einweisung oder Rettungsdiensttransport.		
**Rückeinweisung in die Notaufnahme**	Organisatorisch möglich; keine sektorale Trennung (vgl. sAbbildung 4)	Als Notfalleinweisung (vgl. sAbbildung 4)	Als Notfalleinweisung (vgl. sAbbildung 4)	Als Notfalleinweisung (vgl. sAbbildung 4)
Kurzbeschreibung	Nicht unbedingt als Rückeinweisung verstanden; häufig zur diagnostischen Abklärung oder Therapie; situativ mit Fehleinschätzung (Untertriage) in der Zuweisung begründet.	Zur diagnostischen Abklärung oder Therapie und/oder stationären Einweisung.	Zur diagnostischen Abklärung oder Therapie und/oder stationären Einweisung.	Zur diagnostischen Abklärung oder Therapie und/oder stationären Einweisung. Situativ mit Fehleinschätzung (Untertriage) in der Zuweisung begründet.

Ergänzend zu eTabelle 10 und den teilnehmenden Beobachtungen der Strukturen und Prozesse in den Steuerungsmodellen der NODE-Studie zeigt die folgende Abbildung die potenziellen Versorgungsverläufe von Patient:innen (Studienarm 1 und 2).

eAbbildung 4 Versorgungsverläufe in den Steuerungsmodellen (Studienarm 1 und2) der prospektiven, multizentrischen Mixed-Methods-Kohortenstudie NODE

Anmerkung: Versorgungsverläufe von Patient:innen in der prospektiven, multizentrischen Mixed-Methods-Kohortenstudie NODE nach Studienarm und Studienzentrum bei tatsächlicher Steuerung. In der Studie werden drei Studienarme miteinander verglichen: Studienarm 1 (klinikeigene Ambulanz), Studienarm 2 (KV-Notdienstpraxis) und Studienarm 3 (Standardversorgung mit ärztlicher Einschätzung). Studienziel ist es, die Versorgungseffizienz bzw. das Steuerungspotenzial der Modelle zu ermitteln und zu vergleichen. Die Rekrutierung erfolgte vom 01.06.2023 bis zum 31.08.2024 an sechs Studienzentren in Berlin. Die einzelnen Abbildungen veranschaulichen die möglichen Versorgungsverläufe der Patient:innen in den Steuerungsmodellen: Der rote Track zeigt Verläufe mit Behandlung in der Notaufnahme, der blaue bzw. grüne Track Versorgungsverläufe in der alternativen Versorgungsstruktur mit möglichen Übergängen in die Notaufnahme und zurück. Die Prozessbeobachtungen verliefen parallel zur Rekrutierung für die Primärdatenkohorte.

Onlinematerial: Krüger et al. „Effizienz von Steuerungs- und integrierten Versorgungsmodellen für wenig akute Patient:innen in der Akut- und Notfallversorgung – Ergebnisse einer prospektiven Kohortenstudie“

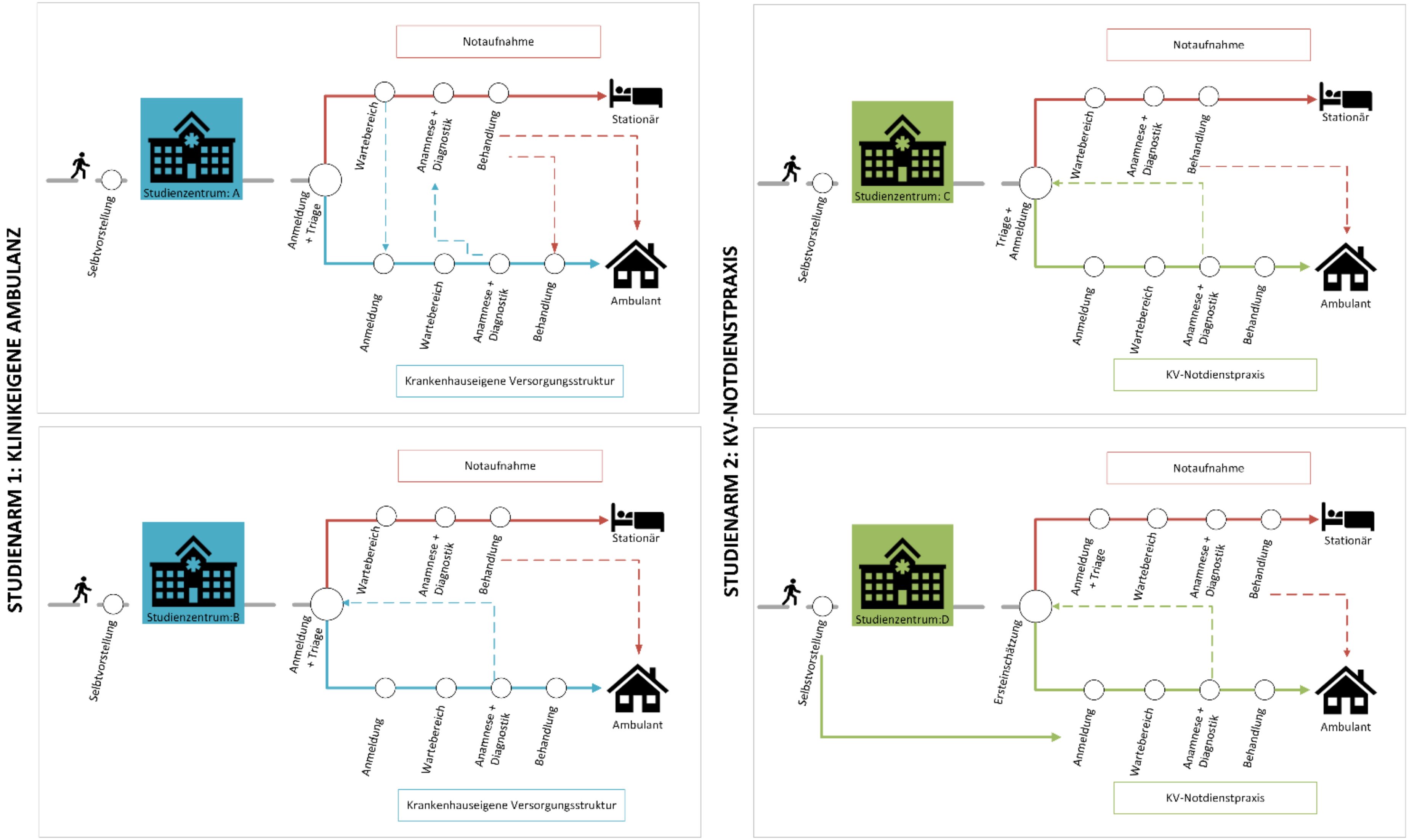

Kosten der Notaufnahmeversorgung (eTabelle 11)

eTabelle 11 Durchschnittliche Kosten pro Patient:in der Behandlung in der Notaufnahme aus Krankenhausperspektive

	Studienarm 1: **Klinikeigene Ambulanz**				Studienarm 2: **KV-Notdienstpraxis**				Studienarm 3: **Standardversorgung**			
	**Klinikeigene Ambulanz** (n=145)		**NA** (n=148)		**KV-Notdienstpraxis** (n=322)		**NA** (n=95)		**Ambulante Praxis** (n=240)		**NA** (n=87)	
	MW	SD	MW	SD	MW	SD	MW	SD	MW	SD	MW	SD
Kosten für die Diagnostik und die ärztlichen Konsile in der Notaufnahme	35 €	89 €	47 €	102 €	3 €	23 €	42 €	91 €	21 €	46 €	85 €	122 €

Anmerkung: In der Studie werden drei Studienarme miteinander verglichen: Studienarm 1 (klinikeigene Ambulanz), Studienarm 2 (KV-Notdienstpraxis) und Studienarm 3 (Standardversorgung mit ärztlicher Einschätzung). Ziel ist es, die Versorgungseffizienz bzw. das Steuerungspotenzial der Modelle zu ermitteln und zu vergleichen. Die Rekrutierung erfolgte vom 01.06.2023 bis zum 31.08.2024 an sechs Studienzentren in Berlin. Darstellung in Bezug auf die initiale Zuweisung in die Alternativstruktur oder Notaufnahme bei tatsächlicher Steuerung (Studienarm 1 und 2) bzw. nach ärztlicher Einschätzung mit Empfehlung zur Versorgung in einer ambulanten Praxis oder der Notaufnahme (Steuerungspotenzial: Studienarm 3); Fallzahl abzüglich n=59 Fälle, die bei Nicht-Verfügbarkeit der Alternativstruktur rekrutiert wurden.

Legende: MW=Mittelwert; SD=Standardabweichung; n=Fallzahl; €=Euro

Verfügbarkeit der Steuerungsmodelle (Studienarm 1 und 2; eTabelle 12)

eTabelle 12 Übersicht über die aggregierte Verfügbarkeit der Steuerungsmodelle (Studienarm 1, 2) in der prospektiven, multizentrischen Mixed-Methods-Kohortenstudie NODE

Anmerkung: In der Studie werden drei Studienarme miteinander verglichen: Studienarm 1 (klinikeigene Ambulanz), Studienarm 2 (KV-Notdienstpraxis) und Studienarm 3 (Standardversorgung mit ärztlicher Einschätzung). Studienziel ist es, die Versorgungseffizienz bzw. das Steuerungspotenzial der Modelle zu ermitteln und zu vergleichen. Die Rekrutierung erfolgte vom 01.06.2023 bis zum 31.08.2024 an sechs Studienzentren in Berlin. Die hier berichtete tatsächliche Verfügbarkeit der Steuerungsmodelle (n=4 Studienzentren) basiert aufgrund unvollständiger Routinedaten im Studienarm 2 auf Angaben aus einem Studienzentrum.

	**Studienarm 1: Klinikeigene Ambulanz**	**Studienarm 2: KV-Notdienstpraxis**
Potenzielle Verfügbarkeitstage (N)	324	205
Tatsächliche Verfügbarkeit (n)	197	205
Anteil (n/N) in %	61%	100%

Kosten aus gesellschaftlicher Perspektive (eTabelle 13)

eTabelle 13 Durchschnittliche Kosten pro Patient:in der Primärdatenkohorte nach Kostenart aus gesellschaftlicher Perspektive für den Nachbeobachtungszeitraum von 30 Tagen

	Studienarm 1: **Klinikeigene Ambulanz**						Studienarm 2: **KV-Notdienstpraxis**						Studienarm 3: **Standardversorgung**					
	**Klinikeigene Ambulanz**			**NA**			**KV-Notdienstpraxis**			**NA**			**Ambulante Praxis**			**NA**		
Kostenarten	N	MW	SD	N	MW	SD	N	MW	SD	N	MW	SD	N	MW	SD	N	MW	SD
Kosten für stationäre Behandlung	59	34 €	263 €	71	1.040 €	4.782 €	140	535 €	2.845 €	45	337 €	1.585 €	102	129 €	941 €	40	860 €	2.867 €
Kosten für teilstationäre Behandlung	59	201 €	1.288 €	72	411 €	2.552 €	138	10 €	112 €	45	44 €	166 €	102	0 €	0 €	39	506 €	2.947 €
Kosten für ambulante Behandlung	58	45 €	89 €	71	48 €	112 €	140	59 €	118 €	47	95 €	197 €	103	65 €	132 €	41	82 €	155 €
Arzneimittelkosten	64	7 €	50 €	79	6 €	35 €	152	27 €	261 €	50	2 €	7 €	112	1 €	7 €	42	23 €	100 €
Heilmittelkosten	64	11 €	40 €	76	14 €	62 €	148	12 €	53 €	49	14 €	51 €	112	17 €	56 €	42	12 €	43 €
Versorgungskosten insgesamt (direkte Kosten)	58	304 €	1.374 €	67	1.614 €	6.265 €	130	638 €	3.144 €	44	508 €	1.645 €	102	215 €	1.019 €	39	1.512 €	5.487 €
Ausfall an Bruttowertschöpfung durch Arbeitsunfähigkeit (indirekte Kosten)	44	1.002 €	2.060 €	46	1.191 €	2.443 €	129	1.287 €	2.175 €	43	1.332 €	2.438 €	86	898 €	1.821 €	35	1.956 €	2.572 €
Gesamtkosten (direkte +indirekte Kosten)	43	1.153 €	2.255 €	41	2.243 €	7.653 €	121	1.889 €	4.512 €	41	1.814 €	3.510 €	86	1.069 €	2.222 €	34	2.693 €	3.814 €

Anmerkung: In der Studie werden drei Studienarme miteinander verglichen: Studienarm 1 (klinikeigene Ambulanz), Studienarm 2 (KV-Notdienstpraxis) und Studienarm 3 (Standardversorgung mit ärztlicher Einschätzung). Ziel ist es, die Versorgungseffizienz bzw. das Steuerungspotenzial der Modelle zu ermitteln und zu vergleichen. Die Rekrutierung erfolgte vom 01.06.2023 bis zum 31.08.2024 an sechs Studienzentren in Berlin. Darstellung in Bezug auf die initiale Zuweisung in die Alternativstruktur oder Notaufnahme bei tatsächlicher Steuerung (Studienarm 1 und 2) bzw. nach ärztlicher Einschätzung mit Empfehlung zur Versorgung in einer ambulanten Praxis oder der Notaufnahme (Steuerungspotenzial: Studienarm 3); Fallzahl abzüglich n=59 Fälle, die bei Nicht-Verfügbarkeit der Alternativstruktur rekrutiert wurden.

Legende: MW=Mittelwert; SD=Standardabweichung; n=Fallzahl; €=Euro

Wartezeit bis zum ersten ärztlichen Kontakt (eAbbildung 4)

Die Grafik zeigt die Wartezeit bis zum ersten ärztlichen Kontakt in Minuten in der Primärdatenkohorte am Tag der Notaufnahmevorstellung (Baseline, t0).

Anmerkung: In der Studie werden drei Studienarme miteinander verglichen: Studienarm 1 (klinikeigene Ambulanz), Studienarm 2 (KV-Notdienstpraxis) und Studienarm 3 (Standardversorgung mit ärztlicher Einschätzung). Studienziel ist es, die Versorgungseffizienz bzw. das Steuerungspotenzial der Modelle zu ermitteln und zu vergleichen. Die Rekrutierung erfolgte vom 01.06.2023 bis zum 31.08.2024 an sechs Studienzentren in Berlin. Darstellung in Bezug auf die initiale Zuweisung in die Alternativstruktur oder Notaufnahme bei tatsächlicher Steuerung (Studienarm 1 und 2) bzw. nach ärztlicher Einschätzung mit Empfehlung zur Versorgung in einer ambulanten Praxis oder der Notaufnahme (Steuerungspotenzial: Studienarm 3); Fallzahl abzüglich n=59 Fälle, die bei Nicht-Verfügbarkeit der Alternativstruktur rekrutiert wurden.

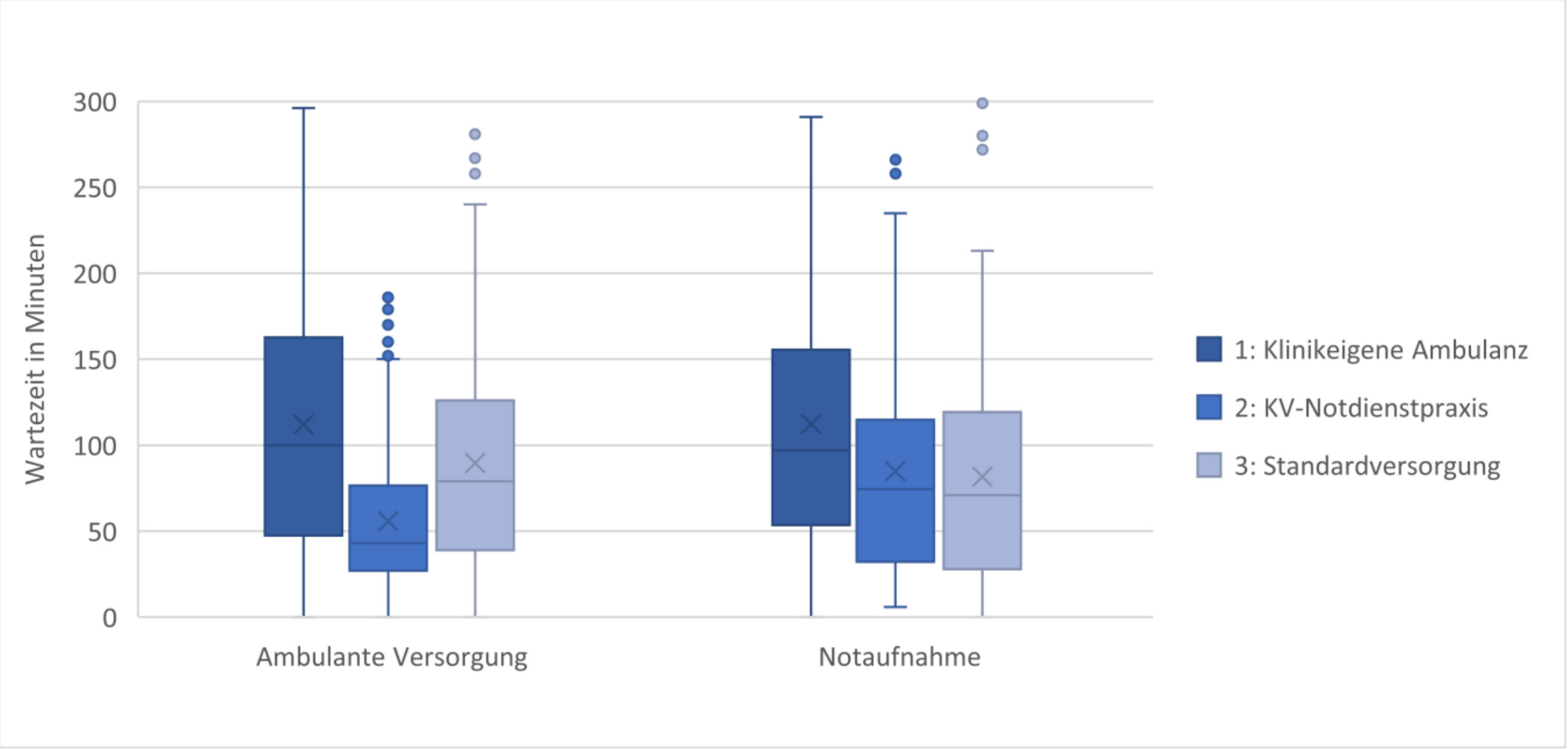

eAbbildung 4 Boxplots der Dauer bis zum ersten ärztlichen Kontakt (Cut-Off bei 300 Minuten) nach initialer Zuweisung in die Alternativstruktur oder Notaufnahme bzw. nach ärztlicher Empfehlung (n=890, nmiss=206)

Zufriedenheit mit der Versorgung (eAbbildung 5)

Die Abbildung zeigt die Versorgungszufriedenheit im Follow-up in NODE nach Studienarm und Versorgungssetting in der Primärdatenkohorte.

eAbbildung 5 Boxplots der Zufriedenheit mit der Versorgung im Follow-up (gemessen mit ZUF-8[1], Skala 8-32; n=451, nmiss=63) in der prospektiven, multizentrischen Mixed-Methods-Kohortenstudie NODE

Anmerkung: In der Studie werden drei Studienarme miteinander verglichen: Studienarm 1 (klinikeigene Ambulanz), Studienarm 2 (KV-Notdienstpraxis) und Studienarm 3 (Standardversorgung mit ärztlicher Einschätzung). Studienziel ist es, die Versorgungseffizienz bzw. das Steuerungspotenzial der Modelle zu ermitteln und zu vergleichen. Die Rekrutierung erfolgte vom 01.06.2023 bis zum 31.08.2024 an sechs Studienzentren in Berlin. Darstellung in Bezug auf die ausschließliche Versorgung in der Alternativstruktur oder der Notaufnahme sowie bei ärztlicher Empfehlung zur Versorgung in der Alternativstruktur bzw. der Notaufnahme.

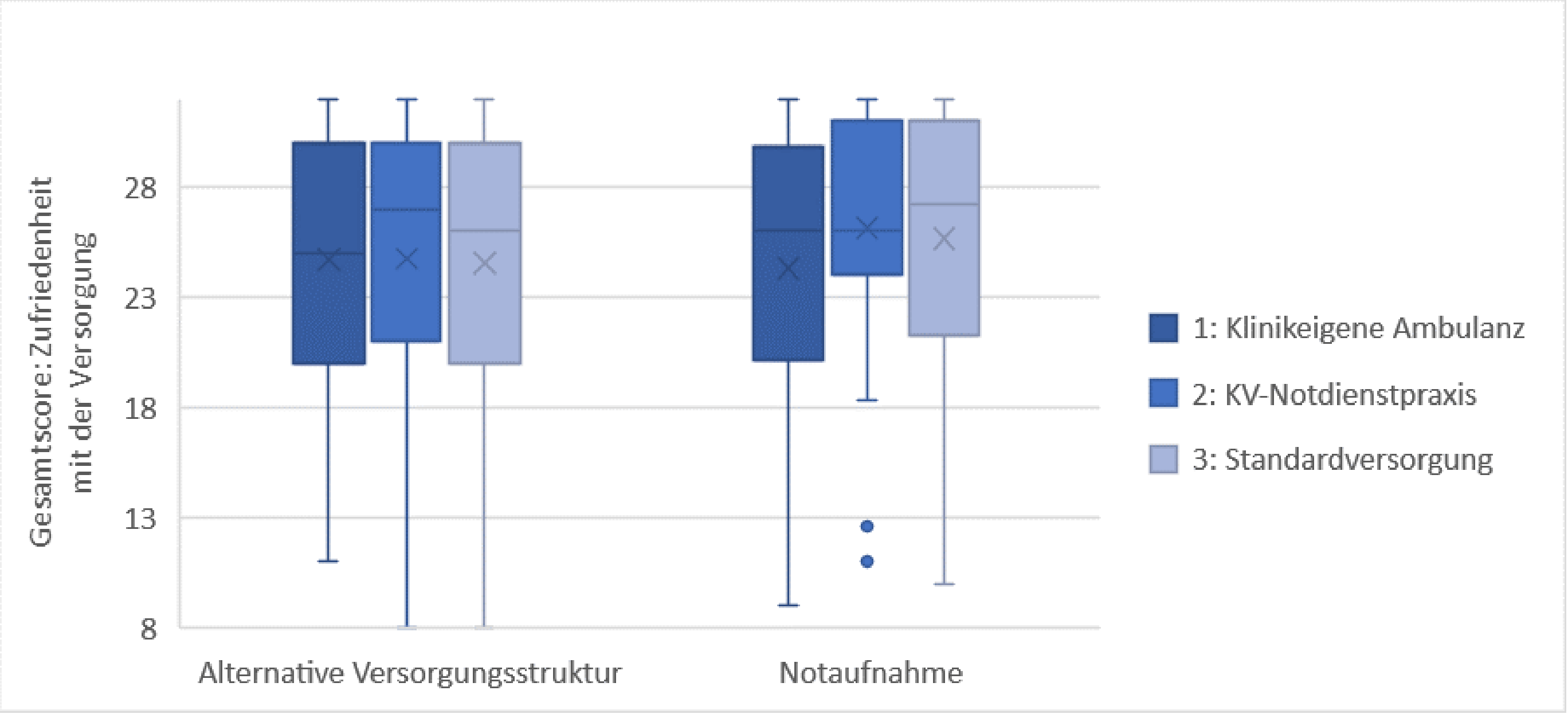

Legende: ZUF-8=Gesamtscore der Patient:innenberichteten Versorgungszufriedenheit (Range: 8-32)[1]

[1] Schmidt, J. and N. Rüdiger (2002). ZUF-8. Fragebogen zur Messung der Patientenzufriedenheit: 392–396.

Stationäre Aufnahmen im Follow-up (eAbbildung 6)

Die Abbildung zeigt die Anteile stationärer Aufnahmen nach Studienarm und Versorgungssetting in der Primärdatenkohorte im 30-Tages-Nachbeobachtungszeitraum.

eAbbildung 6 Stationäre Aufnahmen im Follow-up nach 30 Tagen in der prospektiven, multizentrischen Mixed-Methods-Kohortenstudie NODE

Anmerkung: In der Studie werden drei Studienarme miteinander verglichen: Studienarm 1 (klinikeigene Ambulanz), Studienarm 2 (KV-Notdienstpraxis) und Studienarm 3 (Standardversorgung mit ärztlicher Einschätzung). Studienziel ist es, die Versorgungseffizienz bzw. das Steuerungspotenzial der Modelle zu ermitteln und zu vergleichen. Die Rekrutierung erfolgte vom 01.06.2023 bis zum 31.08.2024 an sechs Studienzentren in Berlin. Darstellung in Bezug auf die ausschließliche Versorgung in der Alternativstruktur oder der Notaufnahme sowie bei ärztlicher Empfehlung zur Versorgung in der Alternativstruktur bzw. der Notaufnahme. Anteil der stationären Aufnahmen insgesamt: 6,3% (n=27; n=432, nmiss=82); 74,1% Bezug zu aktuellen Beschwerden (n=20); 72,0% ungeplant (n=18; nmiss=2); Darstellung in Bezug auf die ausschließliche Versorgung in der Alternativstruktur oder der Notaufnahme sowie bei ärztlicher Empfehlung zur Versorgung in der Alternativstruktur oder der Notaufnahme.

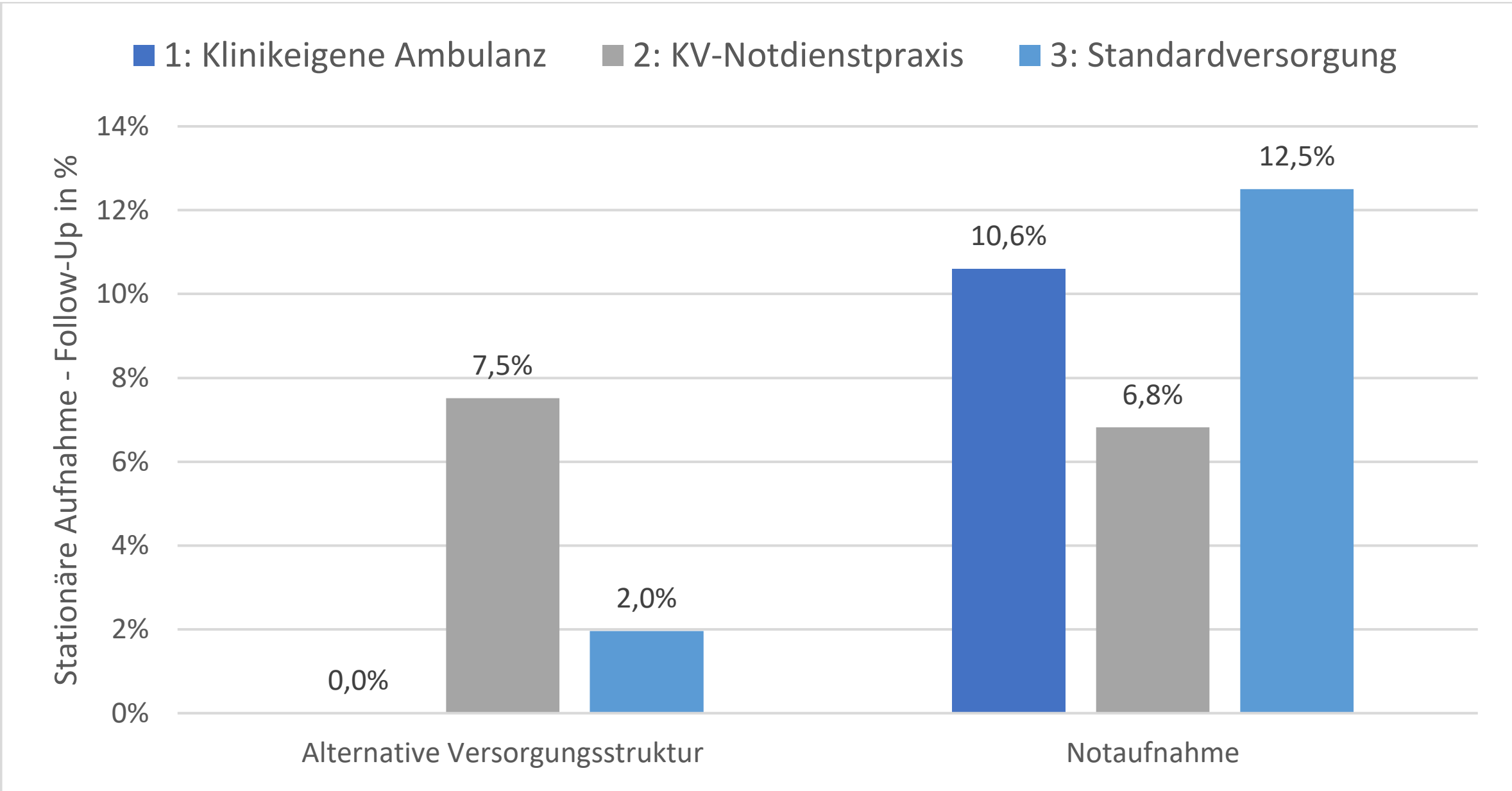

Legende: KV=Kassenärztliche Vereinigung
